# Combining Satellite‐Derived PM_2.5_ Data and a Reduced‐Form Air Quality Model to Support Air Quality Analysis in US Cities

**DOI:** 10.1029/2023GH000788

**Published:** 2023-05-09

**Authors:** Ciaran L. Gallagher, Tracey Holloway, Christopher W. Tessum, Clara M. Jackson, Colleen Heck

**Affiliations:** ^1^ Nelson Institute Center for Sustainability and the Global Environment University of Wisconsin—Madison Madison WI USA; ^2^ Department of Atmospheric and Oceanic Sciences University of Wisconsin—Madison Madison WI USA; ^3^ Department of Civil and Environmental Engineering University of Illinois—Urbana‐Champaign Urbana IL USA

**Keywords:** reduced‐form model, satellite‐derived PM_2.5_, environmental justice, decision‐making, NAAQS, fine particulate matter

## Abstract

Air quality models can support pollution mitigation design by simulating policy scenarios and conducting source contribution analyses. The Intervention Model for Air Pollution (InMAP) is a powerful tool for equitable policy design as its variable resolution grid enables intra‐urban analysis, the scale of which most environmental justice inquiries are levied. However, InMAP underestimates particulate sulfate and overestimates particulate ammonium formation, errors that limit the model's relevance to city‐scale decision‐making. To reduce InMAP's biases and increase its relevancy for urban‐scale analysis, we calculate and apply scaling factors (SFs) based on observational data and advanced models. We consider both satellite‐derived speciated PM_2.5_ from Washington University and ground‐level monitor measurements from the U.S. Environmental Protection Agency, applied with different scaling methodologies. Relative to ground‐monitor data, the unscaled InMAP model fails to meet a normalized mean bias performance goal of <±10% for most of the PM_2.5_ components it simulates (*p*SO_4_: −48%, *p*NO_3_: 8%, *p*NH_4_: 69%), but with city‐specific SFs it achieves the goal benchmarks for every particulate species. Similarly, the normalized mean error performance goal of <35% is not met with the unscaled InMAP model (*p*SO_4_: 53%, *p*NO_3_: 52%, *p*NH_4_: 80%) but is met with the city‐scaling approach (15%–27%). The city‐specific scaling method also improves the *R* ^2^ value from 0.11 to 0.59 (ranging across particulate species) to the range of 0.36–0.76. Scaling increases the percent pollution contribution of electric generating units (EGUs) (nationwide 4%) and non‐EGU point sources (nationwide 6%) and decreases the agriculture sector's contribution (nationwide −6%).

## Key Points

- Applying scaling factors determined with satellite‐derived PM_2.5_ makes the Intervention Model for Air Pollution (InMAP) model suitable and relevant for urban‐scale analysis
- Relative to standard InMAP data, we find higher contributions of electric generating unit (EGU) and non‐EGU point sources to U.S. cities
- Relative to standard InMAP data, we find lower contributions of the agricultural emissions to U.S. cities

## 1 Introduction

Ambient air pollution is the largest environmental health risk and the fifth‐ranked overall risk factor (Cohen et al., 2017). Globally, fine particulate matter (PM_2.5_) has been found responsible for an estimated 4.7 million premature deaths per year (GBD, 2019 Risk Factor Collaborators, 2020). Despite dramatic improvements in air quality in the U.S. due to federal and state regulations, there are still an estimated 121–213 thousand premature deaths stemming from PM_2.5_ pollution each year (Burnett et al., 2018; C.W. Tessum et al., 2019).

A growing body of environmental justice (EJ) literature has documented how communities of color in the United States are exposed to higher concentrations of ambient PM_2.5_ concentrations (Bell & Ebisu, 2012; Clark et al., 2014; Jbaily et al., 2022; Kerr et al., 2021; Mikati et al., 2018; C.W. Tessum et al., 2021). Environmental injustices acutely occur in urban areas, as racial residential segregation and discriminatory placement of environmental burdens collocate vulnerable populations and environmental hazards (Corburn, 2017). Urban air pollution is heterogeneous within cities, census tracts, and even neighborhoods (Apte et al., 2017; Chambliss et al., 2021; Southerland et al., 2021), which influences systematic racial/ethnic exposure disparities (Chambliss et al., 2021).

EJ air pollution research has leveraged ground‐level monitors (Bell & Ebisu, 2012; Miranda et al., 2011) and satellite‐derived data (Castillo et al., 2021; Clark et al., 2014; Kerr et al., 2021), however, these data sources only reveal past ambient air pollution concentrations. Additionally, ground‐level monitor coverage is limited with two‐thirds of U.S. counties not containing a monitor, and coverage has also been found to be unequal across racial/ethnic groups (Miranda et al., 2011). Characterizing exposure is an important step in EJ analysis, but characterization alone will not mitigate or prevent exposure (Van Horne et al., 2022). Rather, progress requires designing equitable pollution mitigation and other policy solutions (Levy, 2021). Models can help design policy solutions by answering decision‐relevant questions, including “what‐if” policy scenarios, and identifying source contributions to air pollution (C. W. Tessum et al., 2019, 2021; Thind et al., 2019). Future policy scenarios and source contribution analyses with air quality models can inform advocates and governments on how to prioritize informed pollution mitigation strategies with limited resources. The fine spatial scale of EJ research inquiries, however, have historically been hard to achieve with chemical transport models. This paper evaluates how integrating a policy‐relevant reduced‐form model with state‐of‐the‐art data products can better support EJ applications.

The InMAP is the only reduced‐form air quality model (RFM) that has spatially detailed results capable of distinguishing ambient air pollution patterns within urban areas (C. W. Tessum et al., 2017), which makes it uniquely appropriate for EJ air pollution research (Gardner‐Frolick et al., 2022). A recent study using InMAP found that coarser grid‐resolution resulted in underpredicting PM_2.5_ pollution exposure and the disparities in exposure between racial/ethnic groups (Paolella et al., 2018).

InMAP deploys a variable resolution grid, which allows the model to have a fine spatial resolution in high population areas and coarser spatial resolution over less populated, rural areas (C. W. Tessum et al., 2017). This approach prioritizes human exposure to air pollution and the subsequent health impacts, while retaining the shorter runtime advantage of RFMs. Like many RFMs, InMAP is a linear model based on a run of a CTM, a methodology that reduces its computational complexity and cost. InMAP has been shown to under‐ and overestimate specific PM_2.5_ species, with accuracy that is suitable for research over large areas (Gilmore et al., 2019; C. W. Tessum et al., 2019; Thind et al., 2019). Over individual cities, however, the model's biases may be too large to support policy design or analysis at the city level.

The concentrations and composition of ambient PM_2.5_ are determined by local and regional emissions as well as meteorology (temperature, precipitation, wind) and geography (Seigneur & Saxena, 1984; Stelson & Seinfeld, 1982). Ambient PM_2.5_ is composed of different PM_2.5_ species, including elemental carbon, organic carbon, sulfate and nitrate compounds, sea salt, and crustal materials. PM_2.5_ can either be directly emitted from anthropogenic sources and natural sources (called primary PM_2.5_) or chemically produced in the atmosphere (called secondary PM_2.5_). In particular, sulfate and nitrate PM_2.5_ are formed by reactions involving nitrogen oxides (NO_x_) and sulfur dioxide (SO_2_) precursor gases respectively. These species are also highly associated with certain sources: particulate ammonium with agriculture and particulate sulfate with coal combustion. InMAP fails to capture the nonlinear chemical reactions that form particulate sulfate PM_2.5_ (*p*SO_4_), resulting in underestimating *p*SO_4_ and overestimating particulate ammonium (*p*NH_4_) formation (C. W. Tessum et al., 2017). As knowing the breakdown of PM_2.5_ species in a given location can inform pollution control strategies, these model biases can affect mitigation decisions.

We assess how to leverage the strengths of InMAP while compensating for the model's weaknesses by integrating satellite and/or monitor data. Bias correction factors derived from monitor and satellite data have been used to improve CTMs (Crooks & Özkaynak, 2014; Friberg et al., 2016; Ghim et al., 2018; Onwukwe & Jackson, 2021; Zhang et al., 2022). Usually, scaling factors (SFs) are applied to emissions, but since InMAP is a linear reduced‐form model, we can scale the concentration results. A thorough investigation into the application of SFs determined by satellite and monitor observations will improve InMAP and its applicability for decisionmakers and EJ community groups. This paper evaluates different approaches to scaling InMAP species of PM_2.5_ with satellite‐derived and ground‐level monitor data.

## 2 Materials and Methods

For this study, we evaluated modeled predictions of speciated PM_2.5_ concentrations of particulate sulfate (*p*SO_4_), particulate nitrate (*p*NO_3_), and particulate ammonium (*p*NH_4_). We estimate annual‐average PM_2.5_ concentrations using the InMAP. InMAP, a reduced‐form air quality model, uses annual‐average chemical, physical, and meteorological inputs derived from a more complex CTM, specifically the Weather Research and Forecasting model coupled with Chemistry (WRF‐Chem) (C. W. Tessum et al., 2017). For our model runs, we used the preprocessed data from a WRF‐Chem run that has been previously described (C. W. Tessum et al., 2017), reflecting 2005 meteorological and chemical data. InMAP also requires annual‐average emissions inputs; we use the most recent input emissions data set available for InMAP, reflecting 2014 emissions. The input emissions were derived from the 2014 National Emissions Inventory (NEI) and initially developed for use in a paper evaluating pollution inequity (C. W. Tessum et al., 2019). We omit international, biogenic, and wildfire emissions, though emissions from prescribed burns and agricultural fires are included.

InMAP reduces computational intensity by employing a variable‐resolution grid. This allows for larger grid cells in rural areas and smaller grid cells in urban, more densely populated areas (down to 1 km × 1 km). The InMAP grid can either be defined by the user of the model or computed by the model itself based on gradients in pollutant concentrations and population density. We employed the former option, a “static” grid, where the grid is defined before the model is run regardless of pollutant concentrations. In our configuration, the largest possible grid cell is 288 km × 288 km and the smallest is 1 km × 1 km; other possible grid cell sizes are 96, 48, 24, 12, 4, and 2 km^2^. To determine the resolution of the grid across the spatial domain, population and population density variables were used in conjunction with U.S. Census Bureau estimates of average population counts for 2011–2015. The population density threshold used for our model runs is 5.5 × 10^−9^ people per kilometer squared and the population threshold is 40,000. In other words, if a given grid cell contains more than 40,000 people or a greater population density than 5.5 × 10^−9^ people per kilometer squared, it was split into smaller cells until 1 km^2^ is reached.

InMAP predictions of *p*SO_4_, *p*NO_3_, and *p*NH_4_ were evaluated against and scaled with satellite‐derived and ground‐level monitor data of speciated PM_2.5_ concentrations. Ground‐level monitor data from U.S. Environmental Protection Agency (EPA) Air Quality System (AQS) was used, specifically the 2014 daily‐averaged PM_2.5_ speciation concentrations (U.S. EPA, 2014).

Ground‐level monitors measure speciated PM_2.5_ via the Chemical Speciation Network as part of the National PM_2.5_ Monitoring Network to evaluate regulatory attainment (*n* = 358 *p*SO_4_, *n* = 351 *p*NO_3_, *n* = 202 *p*NH_4_; U.S. Environmental Protection Agency, 2021). Annual average particulate sulfate, particulate nitrate and particulate ammonium concentrations were calculated for each monitor within InMAP's spatial domain of the contiguous U.S. (*n* = 346 *p*SO_4_, *n* = 339 *p*NO_3_, *n* = 198 *p*NH_4_).

A near surface satellite‐derived PM_2.5_ data product from Washington University North American Regional Estimates provided the satellite 2014 annual mean PM_2.5_, *p*SO_4_, *p*NO_3_, and *p*NH_4_ concentrations (V4.NA.02; van Donkelaar et al., 2019). The publicly available North American Regional Estimates V4.NA.02 data set provides 0.01° × 0.01° gridded estimates of PM_2.5_ total and compositional mass concentrations. Satellites detect the aerosol optical depth (AOD) of the entire column of air by measuring the scattering of light (van Donkelaar et al., 2006). To extrapolate near‐surface PM_2.5_ concentrations, a CTM is used to simulate the relationship between AOD and ground‐level PM_2.5_ and then their data product is also statistically fused with ground‐based monitor data (van Donkelaar et al., 2019). Overall their process introduces two main sources of error: during the measurement of AOD and during the model's inference of the relationship between AOD and PM_2.5_ (Diao et al., 2019). Despite these potential error sources, the Washington University data set has been shown to have high fidelity (van Donkelaar et al., 2019). This data set and other satellite‐derived PM_2.5_ data have been used previously for health applications (Diao et al., 2019). Satellite‐derived data were allocated onto the InMAP grid by calculating the mean PM_2.5_ concentrations of each of the gridded satellite files inside the InMAP grid boxes.

We evaluated different approaches to scaling concentrations with both ground‐level monitor data and satellite data. Approaches to scaling InMAP predicted concentrations include: (a) nationwide (Table S1 in Supporting Information S1); (b) by state (Table S1 in Supporting Information S1); (c) by 5 closest monitors (monitor data only); (d) by grid cell (satellite data only); (e) by urbanized area boundaries (satellite data only, Table S2 in Supporting Information S1). To calculate the SF, we divided the observed average speciated PM_2.5_ concentration (i.e., satellite or ground‐level monitor) by the unscaled InMAP predicted average speciated PM_2.5_ concentration over the place designated (i.e., nationwide, state, grid cell, urbanized area; Equation 1):

(1) $$Scaling\;Factor\;\left(SF\right)=\frac{\sum_{i=1}^{n}O_{i}}{n}÷\frac{\sum_{i=1}^{n}P_{i}}{n}$$

where *P*_*i*_ is predicted InMAP speciated concentrations, *O*_*i*_ is observed (either ground‐level AQS monitor or satellite‐derived speciated concentrations), *n* is the number of grid cells that has both predicted and observed values. Since New Hampshire and Maine did not have a *p*NH_4_ monitor, Vermont's monitor was used to determine the SF. For the fourth scaling approaching, we calculated the centroid of each InMAP grid cell and determined the 5 closest monitors. The fifth scaling approach included cities with a population larger than 350,000 and urbanized area boundaries were obtained from the 2010 Census (Figure S1 in Supporting Information S1, U.S. Census Bureau, 2010). The SFs were then applied to each InMAP grid cell to calculate the scaled InMAP speciation predictions.

InMAP predictions and applied scaling methodologies were evaluated using normalized mean bias (NMB), normalized mean error (NME), mean bias (MB), mean error (ME), and squared Pearson correlation coefficient (*R* ^2^) values where *P*_*i*_ is predicted InMAP speciated concentrations and *O*_*i*_ is observed (i.e., satellite‐derived speciated concentrations or ground‐level monitor data). These statistic metrics are the most commonly used to evaluate model predictions of PM_2.5_ (Simon et al., 2012).

Normalized mean bias (NMB) quantifies the difference between predicted and observed values as a percentage of observed values, and values can range from −100% to +infinity %. NMB is often used to evaluate the level of accuracy considered acceptable for modeling applications; ideally, NMB values should fall between a goal of the closest accuracy any given model can be expected to achieve, and a criteria value. Emery et al. (2017) established benchmark recommendations to evaluate model predictions of daily PM_2.5_ values: for NMB they suggested a goal of <±10% and a criteria of <±30%. Normalized mean error (NME) is similar to NMB, but it uses the absolute difference between predicted and observed values. As such, this metric emphasizes accuracy without consideration of direction (i.e., overestimating or underestimating); values can range from 0 to +infinity. Emery et al. (2017) also made recommendations for NME to have a goal benchmark of <35% and a criteria benchmark of <50%. Coefficient of determination (*R* ^2^) quantifies the amount of variability in model predictions that can be accounted for by the variability in observed values.

InMAP predictions of speciated PM_2.5_ concentrations (unscaled and scaled) more closely align with satellite‐determined estimates when the bias and error metrics approach zero and when the *R* ^2^ value approaches 1. The normalized metrics (NMB, NME) are given as percentages while the absolute metrics (MB, ME) have the same units as the data (μg/m^3^). All metrics were calculated for overall InMAP domain as well as urban, semi‐urban, and rural grid cells.

As this research seeks to evaluate InMAP model bias for application at the city‐level, the model performance statistics were calculated and compared for the whole model output as well as urban, semi‐urban, and rural aggregations (Figure S3 in Supporting Information S1). These delineations are based grid cell size, as InMAP determines grid resolution across the spatial domain based on population thresholds. We define each location type as follows: urban for grid cells 1, 2, and 4 km^2^; semi‐urban for grid cells 12 km^2^; and rural for grid cells 24 km^2^ and larger.

To further demonstrate how the various scaling methodologies could affect urban‐level pollution analysis, we feature 12 illustrative cities (Figure S2 in Supporting Information S1): Louisville/Jefferson County, KY—IN; Chicago, IL—IN; Phoenix—Mesa, AZ; Los Angeles—Long Beach—Anaheim, CA; Pittsburgh, PA; New York—Newark, NY—NJ—CT; New Orleans, LA; Detroit, MI; St. Louis, MO—IL; Las Vegas—Henderson, NV; Kansas City, MO—KS; El Paso, TX—NM. Note these are the names of the urbanized areas as defined by the U.S. Census Bureau. We will use shortened names for ease of communication (i.e., Chicago, IL—IN becomes Chicago, IL).

We additionally evaluate city‐level scaling by calculating the Improvement Ratio to quantitively determine how this scaling methodology affects individual cities or neighborhood level analyses (Equation 2). The improvement ratio is calculated with both satellite‐derived concentrations and ground‐level monitor data as the observed data. If the calculated ratio for a grid cell lies between 0 and 1, then scaled InMAP is closer to the true values than unscaled (i.e., scaling improves the grid cell). If the calculated ratio is >1, then the scaling decreases the agreement with the observed value.

(2) $$Improvement\;Ratio=\left|\frac{Scaled\;InMAP\;predictions-observed\;data}{Unscaled\;InMAP\;predictions-observed\;data}\right|$$

To quantify the source contribution impacts of scaling, we applied the determined SFs to InMAP outputs from running emissions input files separately. These emissions files comprise 15 different emissions sectors, including area fugitive dust, on‐road vehicles, and EGU point sources. As InMAP is a linear model, assuming chemical formation of each species of secondary PM_2.5_ is linear and independent of other species, we can run an emissions file alone without changing its contribution to total PM_2.5_. Then we summed the scaled InMAP predictions and calculated percent contribution from each source.

## 3 Results and Discussion

**Figure 1:**
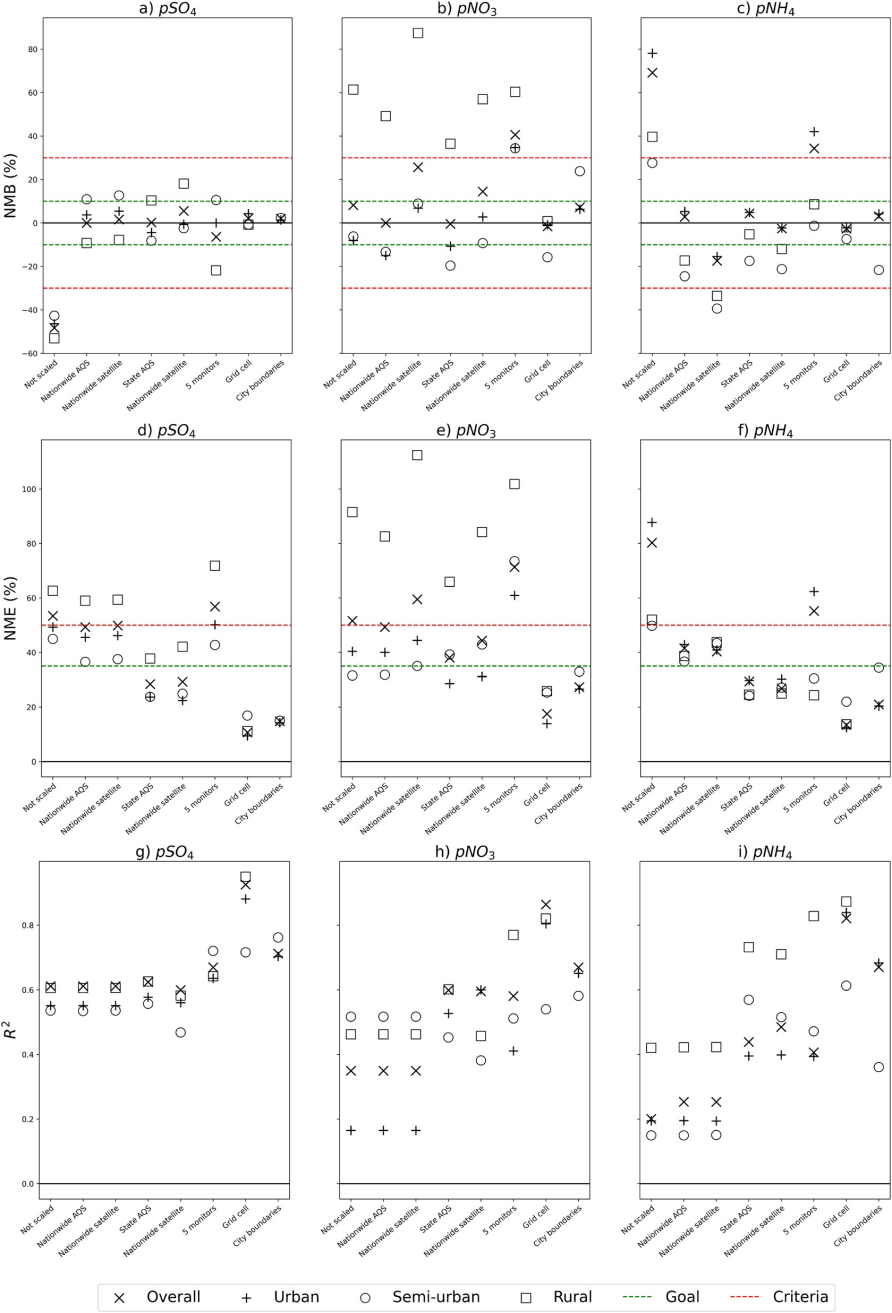
Model performance statistics Normalized Mean Bias (NMB, a–c), Normalized Mean Error (NME, d–f), and coefficient of determination (R^2^, g–i) for overall model domain, urban, semi‐urban, and rural grid cells for each scaling methodology and calculated with ground‐monitor data. From left to right the *x*‐axis, we plot unscaled Intervention Model for Air Pollution predictions and the seven scaling approaches that apply scaling factors determined by Nationwide Air Quality System (AQS), Nationwide satellite, State AQS, State Satellite, 5 monitors, Grid cell, and City Boundaries. NMB and NME graphs also have goal and criteria benchmarks mapped, as recommended by Emery et al. (2017).

The discussed model performance statistics are calculated with ground‐level monitors to align with standard methods of validating chemical transport models (Figure 1, Table S3 in Supporting Information S1). We also calculate model performance statistics with satellite‐derived data as the observed concentration values because satellite‐derived data can indicate model biases across the entire spatial domain of InMAP (Figure S4 and Table S4 in Supporting Information S1). We find statistically significant agreement (alpha = 0.95) across model performance statistics calculated with satellite‐derived and monitor data (*R* ^2^ = 0.83).

We find significant agreement with AQS monitor data and satellite‐derived PM_2.5_ data. We calculated the percent difference between monitor and satellite observations values and R^2^ to be 3% and 0.93 for *p*SO_4_, 7% and 0.86 for *p*NO_3_, and 2% and 0.82 for *p*NH_4_. Not only does this agreement validate evaluating model performance with satellite‐derived data, but it also supports the use of scaling with satellite‐derived data. To note, ground‐based monitor data is an input to the satellite‐derived concentrations (van Donkelaar et al., 2019).

We use grid cell sizes to distinguish between urban, semi, and rural areas, as population and density inform the size of the InMAP grid cells. According to the InMAP population data, rural grid cells account for 25.3% of the population, semi‐urban grid cells account for 16.7% of the population, and urban grid cells account for 58.0% of the population. The percent of rural population aligns with the 2010 census 19.3% (Ratcliffe et al., 2016), which confirms our proxy of grid cell size is able to distinguish between urban and rural locations.

### 3.1 Model Evaluation Metrics for the Different Scaling Methodologies

#### 3.1.1 Unscaled InMAP

Almost all unscaled InMAP predictions fail to meet the NMB performance criteria and goal (overall model domain *p*SO_4_: −48%, *p*NO_3_: 8%, *p*NH_4_: 69%). The overall model domain of InMAP fails to meet both the goal and criteria benchmarks for NME for all particulate species (*p*SO_4_: 53%, *p*NO_3_: 52%, *p*NH_4_: 80%).

We calculate similar R^2^ for unscaled InMAP predictions than C. W. Tessum et al. (2019), which evaluates InMAP output with ground‐monitor observations from 1997 to 2015, although we will compare with their 2014 calculations. The *R* ^2^ for *p*SO_4_ is highest (C. W. Tessum et al., 2019 *R* ^2^ = 0.63, this paper *R* ^2^ = 0.61) and there is worse correlation with *p*NO_3_ (C. W. Tessum et al., 2019 *R* ^2^ = 0.38, this paper *R* ^2^ = 0.35) and *p*NH_4_ (C. W. Tessum et al., 2019 *R* ^2^ = 0.28, this paper *R* ^2^ = 0.20). Notably, we find InMAP predictions of *p*NO_3_ to be better for rural and semi‐urban grid cells but less accurate for urban grid cells and the overall model domain.

Taken together, these evaluation metrics indicate that unscaled InMAP particulate sulfate predictions have moderate spatial correlation to ground‐level monitors and a large model error. Nitrate has a low spatial correlation, but only a slight negative model bias. Ammonium also has a low spatial correlation and a large positive model bias. This unscaled performance agrees with the previous evaluations of the InMAP model (Paolella et al., 2018; C.W. Tessum et al., 2017).

The general trends of InMAP's model biases (underestimating [*p*SO_4_] and overestimating [*p*NH_4_]) can be seen in the difference between InMAP predictions and satellite‐determined PM_2.5_ concentrations: *p*SO_4_ is underestimated across the contiguous U.S. except in the Appalachia Mountains and east Texas (Figure S5a in Supporting Information S1) and *p*NH_4_ is overestimated across the U.S (Figure S7a in Supporting Information S1). In comparison, *p*NO_3_ is mostly overestimated in the eastern part of the U.S., but underestimated in the west and the Great Lakes region (Figure S6a in Supporting Information S1). InMAP's biases and errors stem from the model's reduced complexity nature. The linear equations the govern PM_2.5_ formation limits its accuracy, especially in mountainous regions, possibly due to the complex meteorology in those areas (Thakrar et al., 2022).

#### 3.1.2 Nationwide Scaled InMAP

We apply nationwide SFs determined by AQS monitor and satellite‐derived speciated PM_2.5_ concentrations. AQS‐determined SFs were 1.94 for sulfate, 0.92 for nitrate, and 0.59 for ammonium while satellite‐determined SFs were 1.97 for sulfate, 1.16 for nitrate, and 0.47 for ammonium.

We find that both nationwide SFs yield an improved NMB that falls within the criteria benchmark (<±30%) for particulate sulfate and particulate ammonium across all spatial aggregations (Figure 1). However, the nationwide SF approaches produce disparate results for particulate nitrate: the nationwide AQS SF worsens urban and overall NMB while nationwide satellite SF worsens urban and semi‐urban NMB (Table S3 in Supporting Information S1, Figures 1a–1c).

The nationwide SFs only slightly improve the NME metric. In considering the overall model domain, applying nationwide SFs shifts *p*SO_4_ and *p*NO_3_ under the criteria threshold (<50%) but only the AQS‐derived SF achieves the NME criteria for *p*NH_4_ (Table S3 in Supporting Information S1, Figures 1d–1f). The nationwide scaling methodologies do not improve the R^2^ metric, as applying the same SF across all grid cells does not affect the variability in InMAP predictions (Figures 1g–1i).

In summary, the nationwide SF approaches can correct the large model biases and errors, especially for the more straightforward underestimate of particulate sulfate and overestimate of particulate ammonium. However, the nationwide scaling approach does not improve the model's bias and error in predicting particulate nitrate (NMB: 20%, NME: 48%). As such, we do not recommend applying a nationwide SF to any of the particulate species, especially if model domain includes the Appalachian Mountains, where the model speciated PM_2.5_ predictions diverge from satellite‐derived concentrations (Figures S5–S7 in Supporting Information S1).

#### 3.1.3 State‐Scaled InMAP

We also apply state‐specific SFs determined by ground‐level monitor and satellite‐derived data. Table S1 in Supporting Information S1 report the SFs for each state and species. We find that both state SFs (AQS monitor determined and satellite determined) improve *p*SO_4_ and *p*NH_4_ biases so that the NMB falls within the goal benchmark of <±10% (Figures 1a and 1c). Similar to the nationwide scaling approaches, the state scaling approaches have a more complicated impact on the biases of nitrate. However, this scaling approach does improve *p*NO_3_ enough so that the NMB falls within the criteria benchmark except for rural grid cells for *p*NO_3_ (Figure 1b). The individual SFs for each state also improve the NME so that the model errors for every species and grid cell type achieves the goal benchmark <35% (except for rural *p*NO_3_). Applying state specific SFs improves *R* ^2^ values for particulate nitrate and particulate ammonium but worsens the *R* ^2^ for particulate sulfate (Figure 1g).

In summary, the state SF approaches can correct the large model biases and errors, especially for the more straightforward underestimate of particulate sulfate and overestimate of particulate ammonium. However, the statewide scaling approach does not improve the model's bias and error in predicting particulate nitrate. If InMAP users are interested in investigating state‐specific questions, it can be appropriate to apply state SFs (Jackson et al., 2023). We recommend considering particulate nitrate bias and error for that state to evaluate if this methodology is suitable.

#### 3.1.4 5 Closest Monitors Scaled InMAP

We find that scaling with multiple AQS monitors only marginally improves InMAP model performance. Particulate sulfate biases decreases and the NMB falls within the criteria benchmark for all spatial aggregations and meets the goal benchmark for urban grid cells and overall domain (Figure 1a). However, *p*NO_3_ biases are not corrected and the NMB for *p*NH_4_ only achieves the criteria benchmark for rural and semi‐urban grid cells (Figures 1b and 1c). This scaling methodology does not impact the NME much for *p*SO_4_ or *p*NO_3_, although model errors are improved for *p*NH_4_ (Figures 1d–1f). Applying SFs determined by the 5 closest AQS monitors does improve R^2^ values for all species, although only marginally for *p*SO_4_ (Figures 1g–1i). The average distance of the 5 closest monitors to each grid cell are 113, 170, and 206 km for *p*SO_4_, *p*NO_3_, and *p*NH_4_ respectively. We find no discernible relationship with model improvement and the average distance of 5 monitors, the distance of the closest monitor, or the distance of the furthest monitor.

We do not recommend this approach for the contiguous U.S. However, it could be a suitable methodology if the number and location of regulatory air quality monitors fit the domain of the research question and calculated model performance statistics indicate as such. For example, Jackson et al. (2023) used the data from five speciated monitors in Wisconsin to scale InMAP output, an appropriate approach due to the smaller domain of their research.

#### 3.1.5 Grid Cell Scaled InMAP

We apply SFs determined by satellite‐derived data allocated to the same grid as our InMAP output (shapefile available in SI). The mean SFs were 6.23 for *p*SO_4_, 1.86 for *p*NO_3_, and 0.62 for pNH4 (SF for each city in Table S2 in Supporting Information S1). We find that grid‐scaling improves model bias (<±0.03), model error (<0.17), and the *R* ^2^ (0.82–0.92) for all particulate species and spatial aggregations (Figure 1, Table S3 in Supporting Information S1).

The grid‐scaling methodology is a way to customize every grid cell with satellite‐derived data and requires fewer calculations (in a coding script or ArcGIS). This approach, however, reduces the transparency as it is challenging to calculate and review the SF used across the more than 51,000 grid cells. We recommend using this methodology as we find it to compensate quite well for InMAP's insufficiencies and is relatively easy to apply, despite the large number of SFs applied across the model domain.

#### 3.1.6 City Boundaries Scaled InMAP

Lastly, we apply city‐specific SFs determined by satellite‐derived data for the cities with a population greater than 350,000. Table S2 in Supporting Information S1 reports the SFs for each city and species. For this scaling methodology, we only calculate model performance statistics for cities, not the entire InMAP model domain of the contiguous U.S. (“overall”). Because only the grid cells within the city boundary have been scaled, we omit the rural designation in our model evaluation. We note that this approach approximately yields an implicit population‐weighted SF, as there are more grid cells where the population is larger. Every particulate species and place designation achieves the goal benchmark for NMB and NME with this methodology (Figures 1a–1f). The *R* ^2^ is also improved from the unscaled model and ranges from 0.67 to 0.71 for all grid cells within the scaled cities (Table S3 in Supporting Information S1).

Overall, we find that applying city‐level SFs is an accurate and relevant scaling approach, especially to improve the intra‐urban scale analyses that InMAP was designed to conduct. The grid cell and city boundaries scaling approaches are the only methodologies tested that improve model bias (NME) and model error (NMB) as well as the strength of the relationship between the model and observation (*R* ^2^) for every species (Figure 1, Figure S4, Tables S3 and S4 in Supporting Information S1). We recommend this approach for city‐level analysis as it improves InMAP predictions at a locally‐relevant scale. Furthermore, applying SFs at the city‐level preserves the relationships in the InMAP model while grid‐cell scaling might lead to overfitting. When conducting model scenario runs, it is imperative to retain the mechanistic capabilities of the model, as opposed to results being determined solely by the scaling data set. A city‐level scaling approach improves model bias and error to acceptable levels (Figure 1) without leading to overconfidence in the accuracy of our modeling approach.

**Figure 2:**
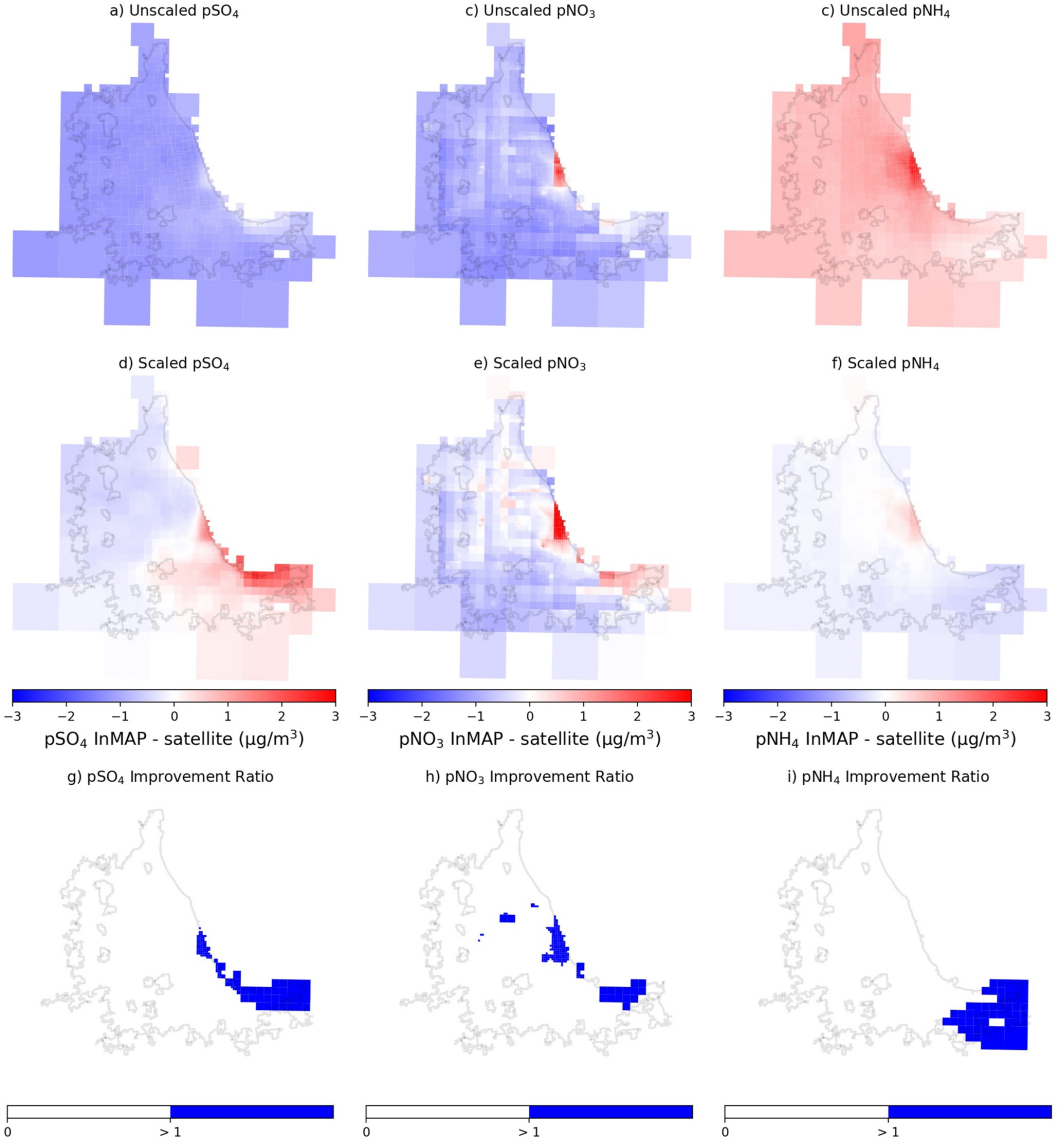
The difference between unscaled Intervention Model for Air Pollution (InMAP) predictions and satellite observations (a–c), the difference between city‐boundary scaled InMAP and satellite observations (d–f), and the ratio between scaled InMAP predictions and satellite observations over Chicago, IL for pSO_4_ (a, d, g), pNO_3_ (b, e, h), and pNH_4_ (c, f, i). (g–i) The Improvement Ratio, where grid cells have improved agreement with satellite observations after scaling if white and decreased agreement if >1 (blue).

**Figure 3:**
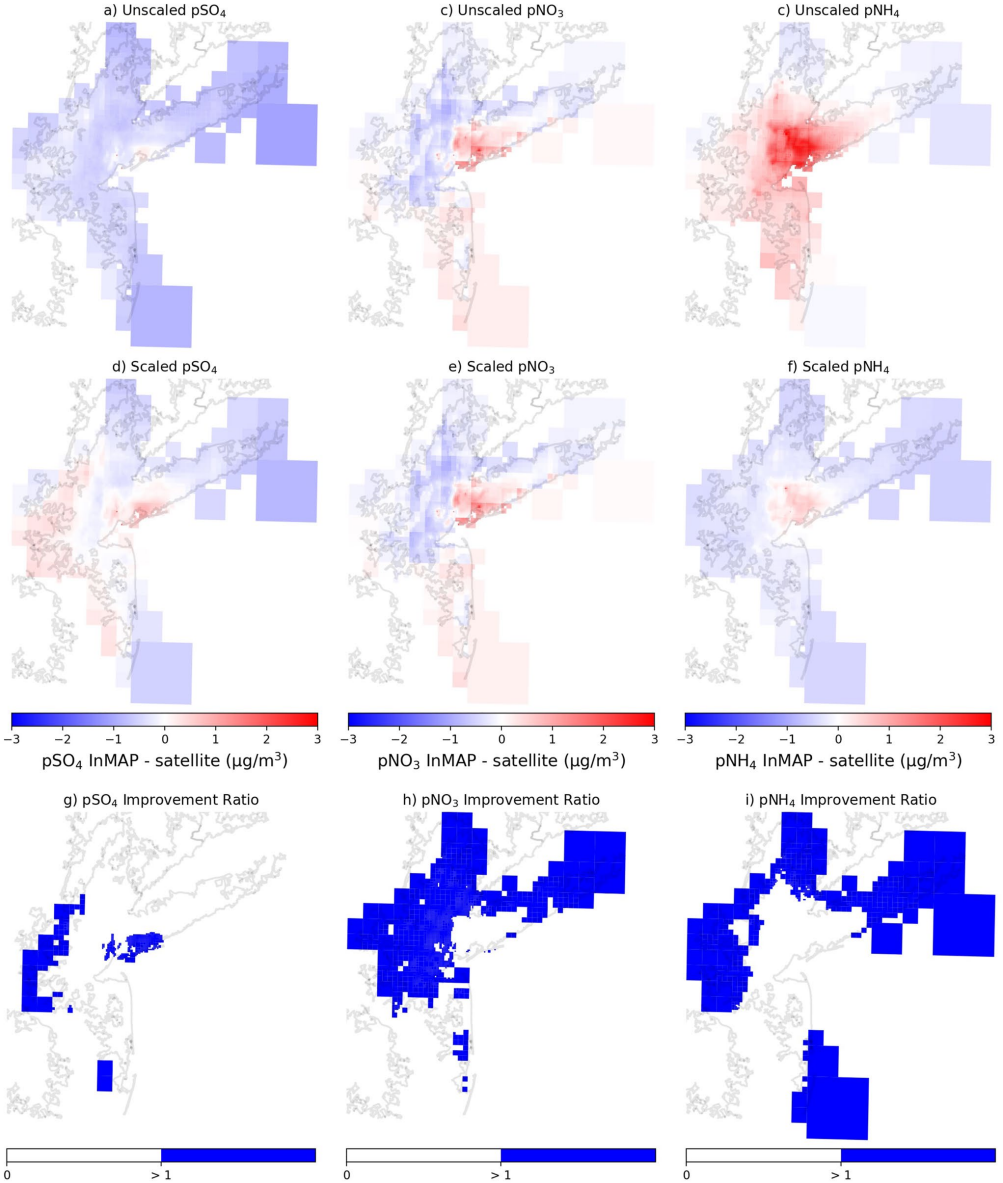
The difference between unscaled Intervention Model for Air Pollution (InMAP) predictions and satellite observations (a–c), the difference between city‐boundary scaled InMAP and satellite observations (d–f), and the ratio between scaled InMAP predictions and satellite observations over New York–Newark, NY–NJ for pSO_4_ (a, d, g), pNO_3_ (b, e, h), and pNH_4_ (c, f, i). (g–i) The Improvement Ratio, where grid cells have improvement agreement with satellite observations after scaling if white and less agreement if >1 (blue).

As we are specifically interested in InMAP's application for intra‐urban analysis, we demonstrate the impact of scaling model predictions at the city‐level in Figures 2 and 3. In these figures, we show the difference between InMAP predictions (unscaled and scaled) and satellite observations as well as a calculated ratio to highlight which grid cells scaling does not improve (i.e., align better with satellite observation). Applying a city‐specific SF affects InMAP model's predictions for Chicago, IL and improves the alignment with satellite‐derived data for almost every grid cell for *p*SO_4_ (Figures 2a, 2d, and 2g), *p*NO_3_ (Figures 2b, 2e, and 2h), and *p*NH_4_ (Figures 2c, 2f, and 2i). For the 12 illustrative cities, scaling improves grid cell alignment with satellite observations 93% for *p*SO_4_, 71% for *p*NO_3_, and 91% for *p*NH_4_ (Table S5 and Figure S10 in Supporting Information S1). When considering the 134 grid cells across the 12 cities with ground‐level monitor data, scaling improves 58% for *p*SO_4_, 63% for *p*NO_3_, and 42% for *p*NH_4_ as determined by the calculated Improvement Ratio (Table S5 and Figure S9 in Supporting Information S1). There are inconsistencies in scaling for individual cities, for example, New York–Newark, NY–NJ (Figure 3) has less alignment with satellite observations and demonstrates two trends: (a) city‐level scaling is least accurate for nitrate; and (b) there is often less alignment between scaled predictions and observation in the outer parts of the city. For many cities, however, this means that scaling is the most accurate in the central, most population dense part of the city, including Chicago and New York–Newark (Figure S11 in Supporting Information S1). When applying a city‐specific SF we recommend investigating these unique discrepancies to determine if this methodology is suitable for the city and research question at hand.

### 3.2 Scaling Impact on InMAP Analysis

#### 3.2.1 Effects of Scaling on Total PM_2.5_ Distribution

**Figure 4:**
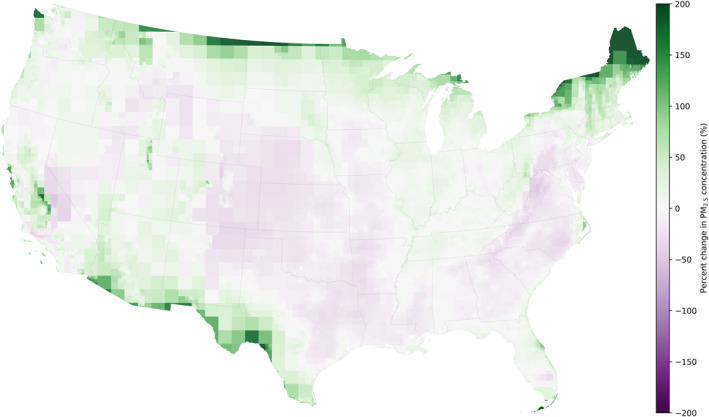
Percent change in total PM_2.5_ concentrations when scaling Intervention Model for Air Pollution predictions with grid cell scaling factors.

Overall scaling increases the average total PM_2.5_ across the continental U.S. by 3% change: unscaled InMAP predictions is 6.96 μg/m^3^ while the scaled (grid cell) total PM_2.5_ is 7.18 μg/m^3^. However, PM_2.5_ concentrations are affected by scaling heterogeneously as shown in Figure 4. Along the edges of the model domain, especially the U.S.‐Mexico and U.S.‐Canada borders, the scaled concentrations are higher than the unscaled InMAP predictions. In contrast, the middle of the country, particularly Midwest and Appalachian Mountains, have lower scaled concentrations compared to the unscaled. Our InMAP model runs do not include international emissions as such, transboundary air pollution from other countries is not included. Figure 4 shows the impact of InMAP only included emissions from the continental U.S. emissions.

#### 3.2.2 Effects of Scaling on Relative Source Contribution

**Figure 5:**
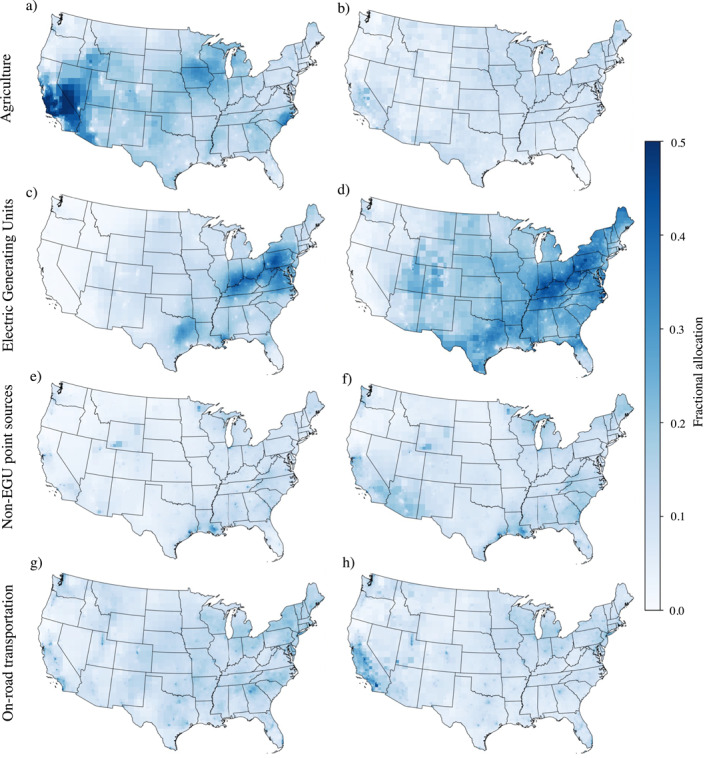
Fractional allocation for the sectors a, b) agriculture, c, d) Electric Generating Units (EGUs), e, f) non‐EGU point sources and g, h) on‐road transportation using unscaled Intervention Model for Air Pollution (InMAP) predictions (left column) and grid‐cell scaled InMAP predictions (right column).

Figure 5 demonstrates the area‐weighted impact that scaling has on InMAP's source contribution analysis for the sectors of agriculture, power plants, and on‐road transportation. Scaling speciated InMAP predictions reduces the impact of the agriculture sector (Figures 5a and 5b) and increases the impact of electricity generating units (i.e., power plants), especially in the northeast (Figures 5c and 5d). These changes in sector source contributions are not surprising as InMAP overestimated particulate ammonium formation which is associated with agriculture emissions and underestimated particulate sulfate formation which is associated with power plant emmisions (Figure S12 in Supporting Information S1). Scaling also increases the relative contribution of non‐EGU (i.e., industry) point sources (Figures 5e and 5f). As industrial sources emit a combination of SO_2_, NO_x_, volatile organic compounds (VOCs) (Figure S12 in Supporting Information S1), this is likely reflecting the scaling increases of *p*SO_4_. On‐road transportation has the largest contribution to air pollution in cities, however, scaling has a more complex impact: it increases transportation's contribution in California cities but decreases it in the eastern part of the U.S (Figures 5g and 5h). The transportation sector primarily emits NO_x_ and VOCs (Figure S12 in Supporting Information S1), and since InMAP overestimates and underestimates *p*NO_3_ formation in different parts of the country (Figure S6 in Supporting Information S1), this complex impact is not surprising. These three trends hold true for large U.S. cities using city‐scaled InMAP predictions (Figure S7 in Supporting Information S1).

#### 3.2.3 Effects of Scaling at the Local Level

**Figure 6:**
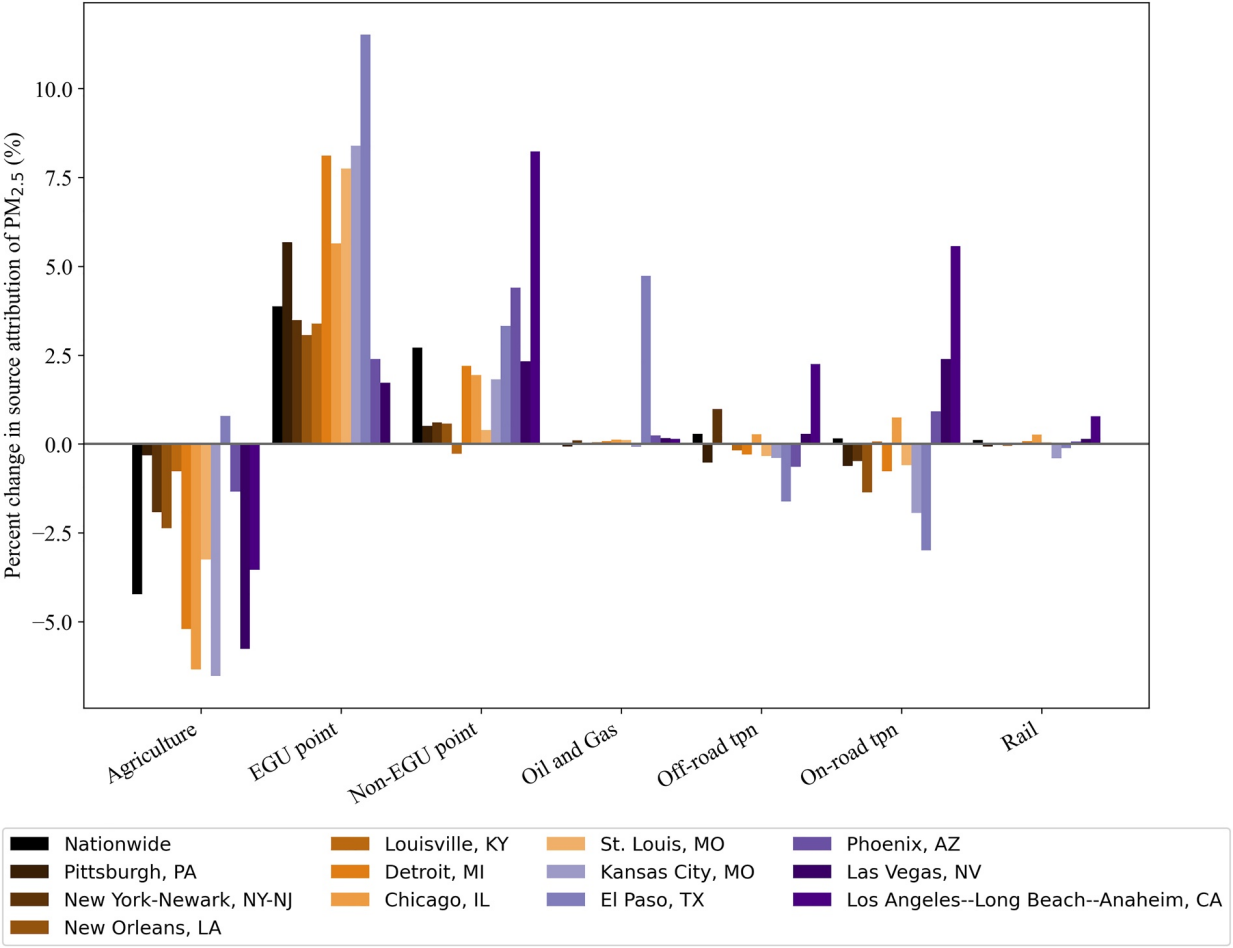
Percent change (%) in pollution contribution from various emissions sources for the contiguous U.S. and 12 cities between unscaled and scaled (city boundaries) Intervention Model for Air Pollution predictions.

To quantify the impact that scaling has on a city‐level source contribution analysis, we calculated the percent change in sector contribution to pollution for the contiguous U.S. and the 12 illustrative cities (Figure 6, Table S6 in Supporting Information S1). For EGU point and non‐EGU point sources, scaling consistently increases the percent pollution contribution, although at different magnitudes. El Paso, TX sees the largest increase in EGU point source contribution (11%, from 2% contribution to 13%) while Los Angeles‐Long Beach‐Anaheim, CA has the smallest increase (<1%). The average increase of EGU pollution contribution for these cities is 5% for EGU and 2% for non‐EGU point sources. Phoenix, AZ has the largest non‐EGU point source pollution contribution increase with scaling at 4%. Scaling consistently decreases the agriculture sector's pollution contribution by 3%. The one exception is El Paso, TX, which sees a slight increase in the agricultural sector's influence on local pollution. Off‐road transportation, on‐road transportation, and rail do not have consistent trends, as these sectors primarily emit NO_x_ (Figure S12 in Supporting Information S1). However, scaling can lead to considerable changes in these sectors. For example, the off‐road transportation contribution increases by 6% in Los Angeles‐Long Beach‐Anaheim, CA but decreases by 3% in El Paso, TX.

Recent papers considering intra‐urban pollution and EJ utilize data collected from mobile pollution monitors (Apte et al., 2017; Chambliss et al., 2021; Southerland et al., 2021) and satellite data products (Castillo et al., 2021; Kerr et al., 2021). While the finer gradient of pollution concentration data of these methods is relevant to EJ issues, alone they are insufficient to determine pollution sources or potential mitigation strategies.

Researchers use two methodologies to conduct source contribution analyses: (a) with measurements and a receptor model, like the U.S. EPA's Positive Matrix Factorization model; and (b) with an emissions inventory and a source‐oriented model, usually a CTM, such as CMAQ or WRF‐Chem. These distinct methods have been deployed at various scales, yield different kinds of results, and have specific embedded methodological limitations. As receptor models estimate source apportionment from the measured mass of PM chemical components, for example, sulfate‐rich PM, this methodology can be limiting in developing policies and mitigation strategies that target specific sectors. Another major limitation is that the receptor model method only works where monitoring data is available. Since cities are highly polluted areas, this source contribution methodology has been employed for cities across the globe (Borlaza et al., 2021; Heo et al., 2017; Silva et al., 2020; Tositti et al., 2014).

Source‐oriented models, in comparison, use emissions inventories and multiple model simulations to consider different sources' impacts on pollution. (In some cases, computational techniques can be used to perform source attribution within a single simulation (Wagstrom et al., 2008). This method can require extensive input data sets (including emissions inventories as well as meteorological data) and computation capabilities. Some research studies have used source‐oriented models at the city level (East et al., 2021; Kheirbek et al., 2016; Ramacher et al., 2020). While past research mostly use CTMs, these models have computational and time intensities that limit their use at the city level. While RFMs address those computation issues, most do not have high enough resolution for this type of analysis. InMAP's resolution over urban areas, however, makes it unique in being a reduced‐form source‐oriented model that can conduct source apportionment at the city level (M. W. Tessum et al., 2022).

As we have demonstrated (Figure 6), scaling particulate sulfate, nitrate, and ammonium concentrations affects the source allocation analysis. InMAP's spatial scale and ability to track the pollution contribution from different sources, once corrected for the model's biases with scaling, allows for an easy‐to‐use methodology that is suitable and relevant to apply to EJ air quality policy questions.

## 4 Conclusions

We have demonstrated the possibility and importance of scaling InMAP speciated particulate concentrations to improve the intra‐urban level analysis. We quantitatively show through multiple model performance metrics (MFB, MFE, and *R* ^2^) that applying grid‐ and city‐specific SFs can improve InMAP predictions. In addition, we have illustrated how scaling increases the percent pollution contribution of EGU point sources (+4% nationwide) and non‐EGU point sources (+6% nationwide) while decreasing the agriculture sector's contribution (−6% nationwide), and that these contribution differences can be even larger for specific cities.

All nationwide, state‐specific, and city‐specific SF are listed in Supporting Information S1 (Tables S3 and S4) so that they can be used by researchers or interested/affected groups when scaling InMAP. Alternately, we have included detailed methodology so that interested parties can identify approrpiate SFs themselves before conducting source contribution analysis or running “what‐if” policy scenarios. While running the InMAP model still requires techinical capacity, we have endeavored to make the model improvement through scaling as transparent, intuitive, and reproducible as possible.

This research strives to leverage the unique capabilities and advantages of different data sources, specifically satellite‐derived data' ability to represent spatially continuous observed pollution concentrations and InMAP's capacity to track pollution sources and model policy scenarios. However, this data fusion might also magnify limitations and uncertainties of each data source. We have investigated a limited number of scaling methodologies, although other bias correction approaches, like neural networks, model parameter tuning, or internal model bias correction, could combine air pollution data products to optimize InMAP model performance.

Our scaling methodologies rely on satellite‐derived concentrations of speciated PM_2.5_ that are estimated through a combination of satellite observations of AOD, CTM model simulation, and ground‐level monitor data. Van Donkelaar et al. (2019) discusses the embedded uncertainties of their geoscience‐statistical hybrid methodology as well as the data product's performance evaluation using 10‐fold cross validation with ground‐based observations, which results in a high fidelity data set (*R* ^2^ = 0.57–0.96). Moreover, we use annual satellite‐derived estimates, which is less prone to daily variability. The satellite data product used has the resolution of 0.01° × 0.01°, which is approximately 1.11 km × 1.11 km at the equator and a similar resolution of the smallest InMAP grid of 1 km × 1 km. The statistical method to allocate the satellite observations onto the InMAP grid map might also introduce errors. However, we also found strong correlation between satellite‐derived estimates on the InMAP grid and monitor observations (*R* ^2^ = 0.82–0.92).

There are some limitations of the performance model metrics. First, we use critiera and goal thresholds that were established for daily PM_2.5_ predictions, not annual PM_2.5_ predictions (Emery et al., 2017). However, to date there are no model performance benchmarks for annual PM_2.5_ predictions. Second, there exists circularity in the data used for scaling and model evaluation. As the satellite‐derived estimates were fused with ground‐level monitor data, scaling with satellite observations and calculating model performance statistics with regulatory monitor data is not an independent assessment. As speciated PM_2.5_ measurements from different sources become available (NSF, 2022), independent assessments of satellite data products and satellite‐scaled model performance will be possible.

Even after scaling, InMAP will have certain policy relevancy limitations. InMAP inputs include preprocessed WRF‐Chem 2005 meteorological data as well as 2014 NEI emissions. While the preprocessed meteorological emissions are from the year 2005, they likely have less impact on the InMAP predictions as the simplified and linear atmospheric chemistry. Calculated model performance for years 1997–2015 changes little, despite the constant inclusion of these 2005 inputs (C. W. Tessum et al., 2019). County‐level NEI emissions are allocated to InMAP grid cells within counties using spatial surrogates, a process that can introduce uncertainty but is the process used by the U.S. Environmental Protection Agency. What might be even more limiting is that the emissions inputs are from the 2014 NEI. As such, the emissions inventory is out of date, particularily for the EGU sector (U.S. Environmental Protection Agency, 2017). Previous research with this model have updated the emissions inputs manually using more up‐to‐date available data, which could be an appropriate approach for an individual city and state analysis (Jackson et al., 2023). Future work could evaluate if applying a second set of satellite‐derived SFs could scale the emissions temporally and further ensure the applicability of the InMAP model to contemporary energy and air pollution inqueries.

While unscaled InMAP model runs might still be appropriate for some nationwide analyses, we recommend using grid‐cell or city‐specific SFs to correct for InMAP's biases when investigating air pollution in cities (individually or as a group). Both of these methodologies use satellite‐derived pollution estimates to calculate the SFs, which enable model bias correction even if cities have a limited number of monitors or monitors placed in locations not representational of the city's pollution burden (i.e., upwind of industry or large thoroughfares). InMAP's modeling capabilities are specifically useful for analyses that track pollution sources or model policy scenarios that relate to emissions sectors as defined by NEI. As InMAP has unprecedented model resolution at the urban level for a reduced‐form model, it is particularly useful to investigate individual cities or compare cities. Paired with U.S. Census demographic data (acquired separately or using the 2012–2016 American Community Survey data at the Census Block Group that has been allocated to the model grid), InMAP can answer questions relating to pollution exposure inequalities across race, ethnicity, income, and other vulnerability characteristics.

Despite InMAP's capacity for urban‐scale and EJ analysis, it is not suitable for all EJ or city‐specific air quality research questions. InMAP's smallest grid cell is 1 km × 1 km, while an analysis of U.S. Census Bureau data estimated neighborhood sizes to range from 0.52 to 1.06 km (Donaldson, 2013). Even when InMAP's grid cells match a neighborhood's size, they may not map neatly onto city neighborhoods that are relevant for EJ inquieries (i.e., investigating the legacy of redlined neighborhoods). Additionally, if end users are interested only in total PM_2.5_ concentration gradients between neighborhoods, satellite observations or low‐cost monitors can provide data at a smaller spatial scale that could be better related to neighborhood boundaries. Moverover, some community‐driven science questions that relate to the impacts of one infrastructure project (e.g., an industrial power plant, a bus depot) may not have enough magnitude of change in emissions to result in a meaningful change to annual average PM_2.5_ concentrations. Finally, as a reduced‐form model, InMAP can only model changes to PM_2.5_ concentrations so investigating other species, including ozone, would require the use of a CTM, satellite observations, or monitor data.

Our intention was to improve InMAP PM_2.5_ predictions at the urban scale through scaling, and thus more appropriate for answering research questions and supporting policy development. This research presents a path for satellite and monitor data to support EJ research application that leverages the limitations and strengths of the different data sources (e.g., ground‐monitor data and satellite‐derived data products) and air quality models.

## Conflict of Interest

The authors declare no conflicts of interest relevant to this study.

## Supporting information

Supporting Information S1

## Data Availability

InMAP is available for download at https://inmap.run/docs/quickstart/. EPA AQS PM_2.5_ monitor data are available at https://aqs.epa.gov/aqsweb/airdata/download_files.html. The near surface satellite‐derived PM_2.5_ data product is available for download at https://sites.wustl.edu/acag/datasets/surface-pm2-5/. Additional Supporting Information S1 available at https://doi.org/10.5281/zenodo.7857094.
