# Supplementary material for: Combining Satellite‐Derived PM2.5 Data and a Reduced‐Form Air Quality Model to Support Air Quality Analysis in US Cities
Source: Geohealth. 2023 May 9;7(5):e2023GH000788. doi: 10.1029/2023GH000788 (PMC10169548; doi:10.1029/2023GH000788)
Supplement: Supplementary file 1 — Supporting Information S1 [file GH2-7-e2023GH000788-s001.pdf]

AGU PUBLICATIONS

AGU GeoHealth

Supporting Information for

Combining satellite-derived $PM_{2.5}$ data and a reduced-form air quality model to support air quality analysis in US cities

Ciaran L. Gallagher[1], Tracey Holloway[1,2], Christopher W. Tessum[3], Clara M. Jackson[1], Colleen Heck[1]

[1]Nelson Institute Center for Sustainability and the Global Environment, University of Wisconsin—Madison, Madison, Wisconsin 53705

[2]Department of Atmospheric and Oceanic Sciences, University of Wisconsin—Madison, Madison, Wisconsin 53705

[3]Department of Civil and Environmental Engineering, University of Illinois—Urbana-Champaign, Urbana, IL 61801

Contents of this file

Equations S1 to S5
Figures S1 to S12
Tables S1 to S6

Additional Supporting Information available at https://doi.org/10.5281/zenodo.7857094.

CSV file of Tables S1 to S2
SHP file (and other associate shapefile files) for grid-cell scaling factors

Equations S1-5 were used to evaluate model performance:

$$\text{(S1) Normalized Mean Bias (NMB)} = 100\% \times \sum_{i=1}^{n} \frac{\sum(P_i - O_i)}{\sum O_i}$$

$$\text{(S2) Normalized Mean Error (NME)} = 100\% \times \sum_{i=1}^{n} \frac{\sum|(P_i - O_i)|}{\sum O_i}$$

$$\text{(S3) Mean Bias (MB)} = \frac{1}{n}\sum_{i=1}^{n}(P_i - O_i)$$

$$\text{(S4) Mean Error (ME)} = \frac{1}{n}\sum_{i=1}^{n}|P_i - O_i|$$

$$\text{(S5) Coefficient of determination } (R^2) = \left(\frac{\sum_1^n ((P_i - \bar{P}) \times (O_i - \bar{O}))}{\sqrt{\sum_1^n (P_i - \bar{P})^2 \ \sum_1^n (O_i - \bar{O})^2}}\right)^2$$

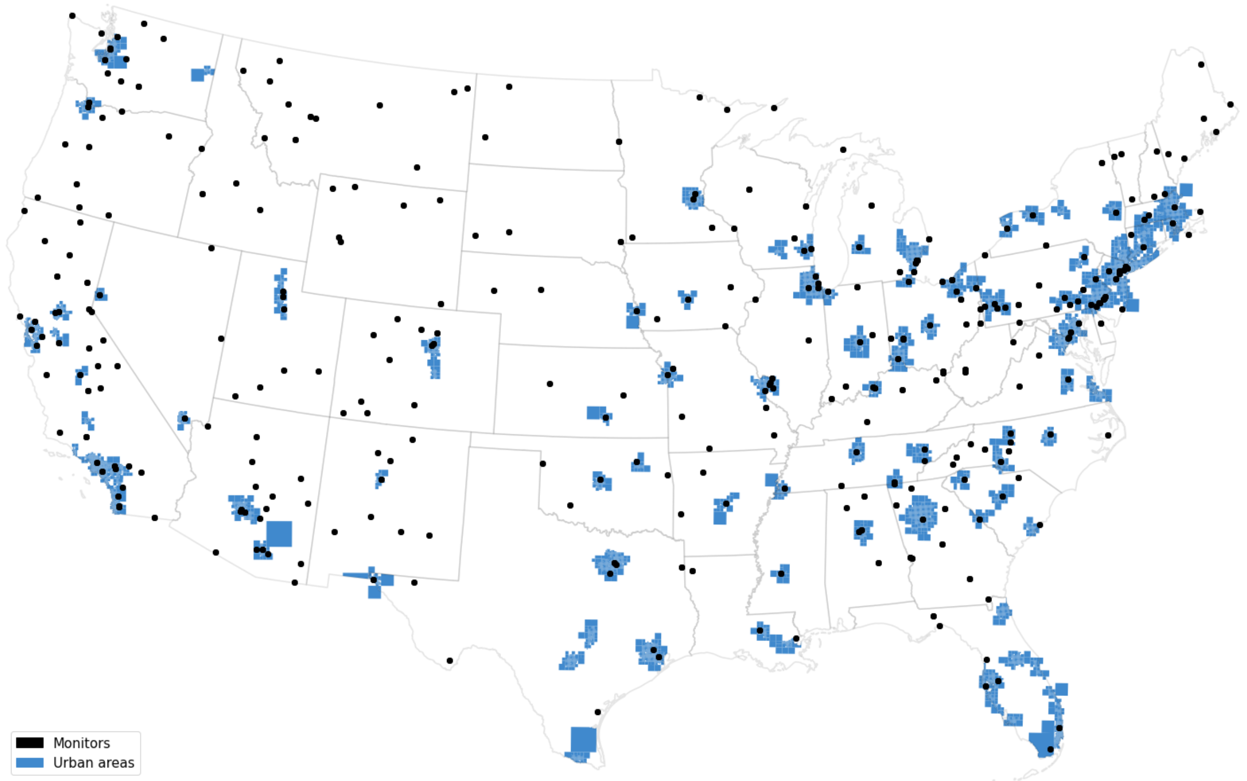

Figure S1. Urban areas with a population greater than 350,000 that are included in the city boundary scaling methodology as well as the locations of the AQS speciation monitors. Not all monitors have data for all three species. Areas highlighted in blue boxes and monitors in blue circles.

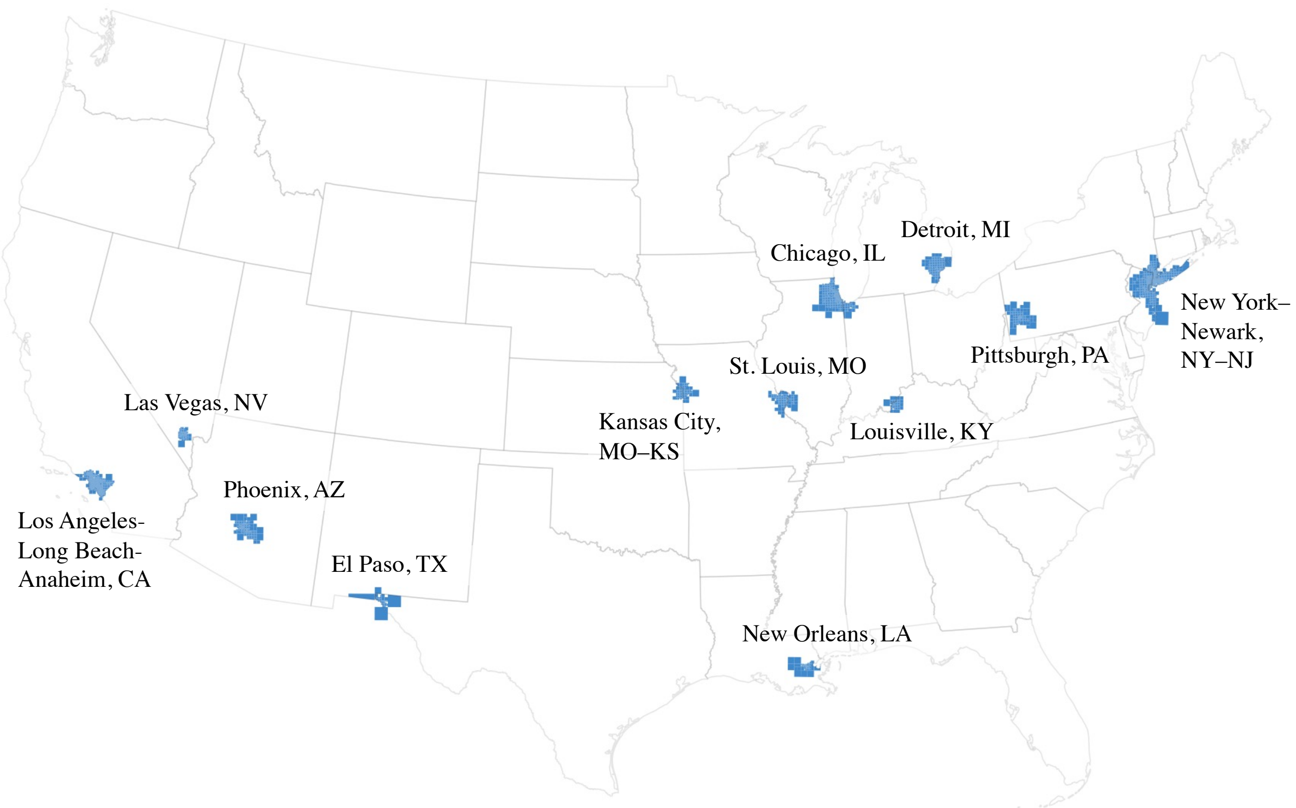

Figure S2. The twelve illustrative cities we highlight, which include Louisville, KY, Chicago, IL, Phoenix, AZ, Los Angeles-Long Beach-Anaheim, CA, Pittsburgh, PA, New York-Newark, NY-NJ, New Orleans, LA, Detroit, MI, St. Louis, MO, Las Vegas, NV, Kansas City, MO-KS, El Paso, TX.

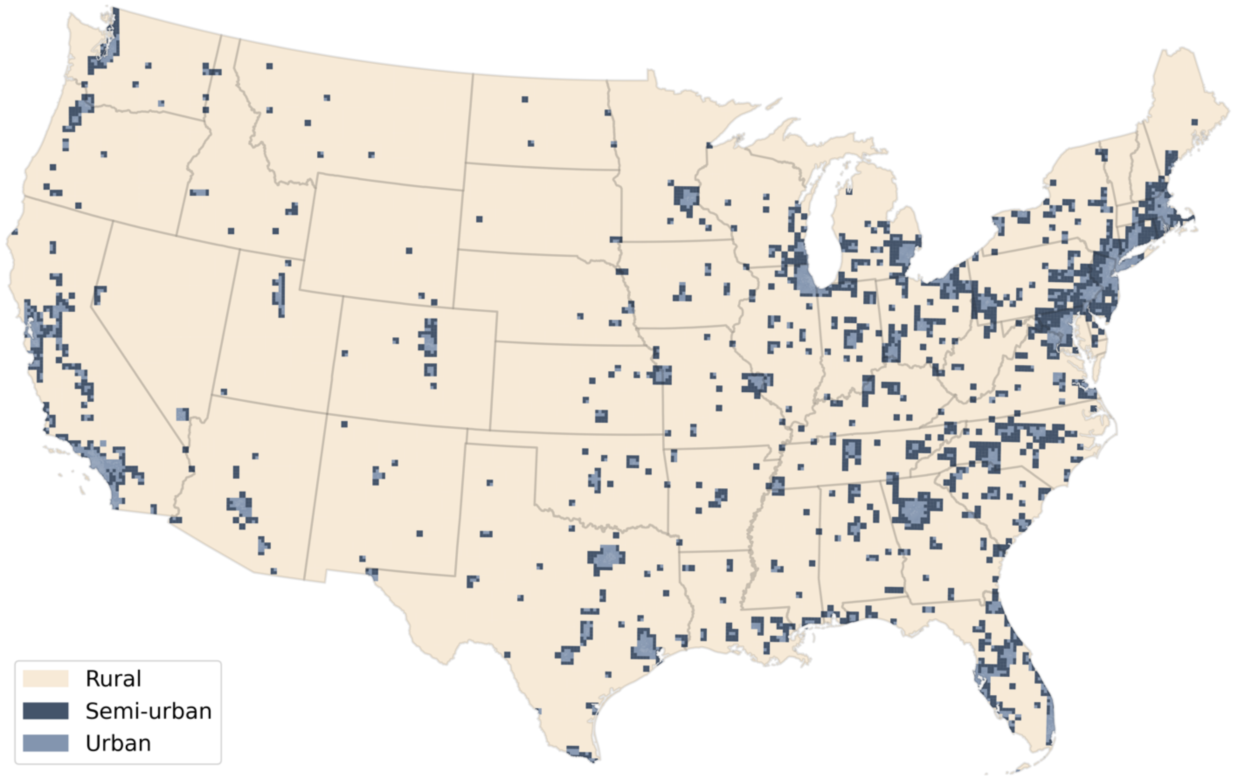

Figure S3. Urban, semi-urban, and rural designations used for the performance metrics disaggregation.

**Domain**	**AQS SF Sulfate**	**AQS SF Nitrate**	**AQS SF Ammonium**	**Satellite SF Sulfate**	**Satellite SF Nitrate**	**Satellite SF Ammonium**
Nationwide	1.94	0.92	0.59	1.97	1.16	0.47
Alabama	1.74	0.60	0.61	1.78	0.48	0.51
Arizona	12.76	2.32	1.59	13.86	2.56	0.60
Arkansas	1.99	0.36	0.66	2.19	0.37	0.57
California	9.23	1.65	0.29	12.80	2.81	0.30
Colorado	1.98	0.59	0.80	2.70	1.07	0.44
Connecticut	1.84	1.11	0.93	2.16	1.37	1.07
Delaware	1.05	1.05	0.74	1.00	0.69	0.57
Florida	4.44	1.46	0.40	3.56	1.45	0.25
Georgia	2.09	0.36	0.38	2.38	0.35	0.39
Idaho	13.78	1.91	1.31	13.80	1.60	0.68
Illinois	2.19	1.28	0.51	2.26	1.39	0.53
Indiana	1.52	1.06	0.83	1.55	1.10	0.83
Iowa	3.39	1.08	0.54	3.49	1.07	0.49
Kansas	2.46	0.66	0.58	2.62	0.63	0.47
Kentucky	1.38	0.78	0.81	1.39	0.92	0.88
Louisiana	1.48	0.57	0.61	1.24	0.48	0.46
Maine	4.01	0.67	1.75	4.67	0.93	1.47
Maryland	0.92	0.69	0.60	0.97	0.83	0.69
Massachusetts	2.58	0.92	0.89	2.77	1.01	0.91
Michigan	2.13	1.13	0.68	2.35	1.25	0.66
Minnesota	3.72	1.13	0.69	4.20	1.33	0.63
Mississippi	1.57	0.56	0.56	1.68	0.48	0.55
Missouri	2.31	0.79	0.69	2.29	0.84	0.62
Montana	10.40	1.12	0.80	9.36	1.06	0.83
Nebraska	2.69	0.63	0.67	3.83	0.87	0.55
Nevada	6.60	1.95	0.21	10.34	4.17	0.25
New Hampshire	3.03	0.85	1.27	3.58	1.38	1.28
New Jersey	1.27	1.08	0.43	1.35	1.14	0.42
New Mexico	5.62	0.90	0.65	5.56	1.04	0.56
New York	1.65	0.89	0.46	1.66	0.90	0.49
North Carolina	1.19	0.41	0.41	1.45	0.39	0.42
North Dakota	4.11	0.83	1.84	3.37	0.86	1.29
Ohio	1.62	0.98	0.96	1.60	0.92	0.90
Oklahoma	2.02	0.51	0.58	2.13	0.55	0.53
Oregon	8.54	0.28	0.46	10.59	0.94	0.44
Pennsylvania	1.18	0.82	0.77	1.17	0.78	0.74
Rhode Island	2.31	0.92	0.68	2.37	0.88	0.88
South Carolina	1.79	0.49	0.44	1.93	0.40	0.39

South Dakota	3.23	0.57	0.98	4.08	0.89	0.72
Tennessee	2.16	0.74	0.74	2.28	0.71	0.69
Texas	1.55	0.28	0.65	1.49	0.57	0.55
Utah	14.51	3.53	1.26	16.33	4.43	0.70
Vermont	5.55	0.90	1.71	5.93	0.94	1.81
Virginia	0.91	0.56	0.64	0.98	0.75	0.65
Washington	9.83	1.07	0.74	10.38	1.09	0.58
West Virginia	1.65	1.15	1.53	1.41	0.84	1.11
Wisconsin	3.04	1.31	0.57	3.04	1.33	0.57
Wyoming	3.11	0.44	0.50	3.50	0.44	0.31

Table S1. The monitor and satellite determined scaling factors for nationwide and state specific scaling methodologies.

City	Population	SF Sulfate	SF Nitrate	SF Ammonium
Akron, OH	569,499	2.05	0.85	0.90
Albany--Schenectady, NY	594,962	2.46	1.19	0.88
Albuquerque, NM	741,318	6.78	2.24	0.49
Allentown, PA--NJ	664,651	1.21	0.88	0.89
Atlanta, GA	4,515,419	2.53	0.38	0.45
Augusta-Richmond County, GA--SC	386,787	2.13	0.29	0.32
Austin, TX	1,362,416	1.67	0.60	0.60
Bakersfield, CA	523,994	42.09	2.51	0.66
Baltimore, MD	2,203,663	0.92	0.90	0.66
Baton Rouge, LA	594,309	1.21	0.38	0.58
Birmingham, AL	749,495	1.69	0.67	0.50
Boston, MA--NH--RI	4,181,019	2.87	0.99	0.85
Bridgeport--Stamford, CT--NY	923,311	1.93	1.57	1.02
Buffalo, NY	935,906	2.81	1.07	1.54
Cape Coral, FL	530,290	4.49	1.29	0.10
Charleston--North Charleston, SC	548,404	2.28	0.61	0.37
Charlotte, NC--SC	1,249,442	1.75	0.35	0.52
Chattanooga, TN--GA	381,112	2.29	0.54	0.55
Chicago, IL--IN	8,608,208	2.23	1.42	0.50
Cincinnati, OH--KY--IN	1,624,827	1.16	1.17	1.00
Cleveland, OH	1,780,673	2.31	0.91	1.13
Colorado Springs, CO	559,409	1.30	0.90	0.18
Columbia, SC	549,777	1.88	0.51	0.43
Columbus, OH	1,368,035	1.38	0.96	0.74
Concord, CA	615,968	7.72	2.72	0.46
Dallas--Fort Worth--Arlington, TX	5,121,892	1.12	0.63	0.48
Dayton, OH	724,091	1.35	1.10	0.90
Denton--Lewisville, TX	366,174	1.27	0.66	0.55
Denver--Aurora, CO	2,374,203	3.49	1.32	0.54

Des Moines, IA	450,070	3.26	1.01	0.49
Detroit, MI	3,734,090	2.42	1.33	0.62
El Paso, TX--NM	803,086	13.61	3.23	1.80
Flint, MI	356,218	2.88	1.46	0.78
Fresno, CA	654,628	40.55	3.18	1.16
Grand Rapids, MI	569,935	2.37	1.20	0.76
Greenville, SC	400,492	1.57	0.41	0.39
Harrisburg, PA	444,474	0.86	0.59	0.93
Hartford, CT	924,859	2.37	1.37	0.93
Houston, TX	4,944,332	1.53	0.45	0.58
Indianapolis, IN	1,487,483	1.50	1.13	0.90
Jackson, MS	351,478	2.28	0.48	0.54
Jacksonville, FL	1,065,219	2.02	0.94	0.40
Kansas City, MO--KS	1,519,417	2.60	0.85	0.54
Knoxville, TN	558,696	2.71	0.79	0.70
Lancaster, PA	402,004	1.00	0.43	1.07
Las Vegas--Henderson, NV	1,886,011	13.19	5.83	0.31
Little Rock, AR	431,388	2.55	0.50	0.59
Los Angeles--Long Beach--Anaheim, CA	12,150,996	10.37	3.23	0.27
Louisville/Jefferson County, KY--IN	972,546	1.30	1.22	0.99
Madison, WI	401,661	3.56	1.23	0.56
McAllen, TX	728,825	7.15	0.74	0.97
Memphis, TN--MS--AR	1,060,061	2.87	0.62	0.60
Miami, FL	5,502,379	12.58	2.42	0.19
Milwaukee, WI	1,376,476	2.99	1.42	0.53
Minneapolis--St. Paul, MN--WI	2,650,890	4.59	1.42	0.66
Mission Viejo--Lake Forest--San Clemente, CA	583,681	9.98	2.67	0.26
Modesto, CA	358,172	10.53	2.04	0.44
Murrieta--Temecula--Menifee, CA	441,546	16.20	2.10	0.38
Nashville-Davidson, TN	969,587	2.08	0.88	0.83
New Haven, CT	562,839	2.15	1.34	1.11
New Orleans, LA	899,703	1.22	0.74	0.52
New York--Newark, NY--NJ--CT	18,351,295	1.40	0.98	0.37
Ogden--Layton, UT	546,026	20.34	4.41	0.88
Oklahoma City, OK	861,505	2.44	0.57	0.46
Omaha, NE--IA	725,008	4.10	0.99	0.62
Orlando, FL	1,510,516	4.07	1.45	0.36
Oxnard, CA	367,260	25.22	3.63	0.27
Palm Bay--Melbourne, FL	452,791	7.38	2.47	0.31
Philadelphia, PA--NJ--DE--MD	5,441,567	1.05	0.90	0.45
Phoenix--Mesa, AZ	3,629,114	15.44	2.39	0.64
Pittsburgh, PA	1,733,853	1.64	1.28	1.11

Port St. Lucie, FL	376,047	9.54	3.58	0.23
Portland, OR--WA	1,849,898	11.39	0.97	0.38
Poughkeepsie--Newburgh, NY--NJ	423,566	2.12	1.54	1.56
Providence, RI--MA	1,190,956	2.32	0.89	0.91
Provo--Orem, UT	482,819	21.29	3.79	0.95
Raleigh, NC	884,891	1.32	0.44	0.52
Reno, NV--CA	392,141	4.58	1.82	0.11
Richmond, VA	953,556	1.12	0.71	0.62
Riverside--San Bernardino, CA	1,932,666	18.58	3.00	0.35
Rochester, NY	720,572	2.43	1.26	1.28
Sacramento, CA	1,723,634	13.80	2.03	0.35
Salt Lake City--West Valley City, UT	1,021,243	15.89	5.92	0.60
San Antonio, TX	1,758,210	2.75	0.88	0.74
San Diego, CA	2,956,746	20.96	1.66	0.32
San Francisco--Oakland, CA	3,281,212	14.24	3.09	0.28
San Jose, CA	1,664,496	14.09	2.71	0.24
Sarasota--Bradenton, FL	643,260	2.35	1.96	0.23
Scranton, PA	381,502	1.69	1.00	1.45
Seattle, WA	3,059,393	11.51	1.03	0.47
Spokane, WA	387,847	17.58	1.93	1.52
Springfield, MA--CT	621,300	2.72	1.47	1.09
St. Louis, MO--IL	2,150,706	2.13	1.24	0.74
Stockton, CA	370,583	8.95	2.44	0.35
Syracuse, NY	412,317	2.52	1.11	1.06
Tampa--St. Petersburg, FL	2,441,770	2.22	1.38	0.36
Toledo, OH--MI	507,643	2.19	1.10	0.71
Tucson, AZ	843,168	10.31	9.10	0.48
Tulsa, OK	655,479	1.97	0.74	0.77
Virginia Beach, VA	1,439,666	1.42	1.01	0.77
Washington, DC--VA--MD	4,586,770	1.00	0.90	0.65
Wichita, KS	472,870	2.84	0.61	0.53
Winston-Salem, NC	391,024	1.32	0.70	0.56
Worcester, MA--CT	486,514	2.81	0.81	1.17
Youngstown, OH--PA	387,550	1.87	0.87	1.11

Table S2. The population and city-boundary determined scaling factors for each city larger than 350,000 people.

Type of Analysis		Overall					Urban				
Scaling type	Species	NMB	NME	MB	ME	R2	NMB	NME	MB	ME	R2
Unscaled	SO4	-0.48	0.53	-0.60	0.66	0.61	-0.46	0.49	-0.73	0.77	0.55
	NO3	0.08	0.52	0.08	0.48	0.35	-0.08	0.40	-0.11	0.57	0.16
	NH4	0.69	0.80	0.49	0.57	0.20	0.78	0.88	0.57	0.64	0.19
Nationwide SF (AQS)	SO4	0.00	0.49	0.00	0.61	0.61	0.04	0.46	0.06	0.72	0.55
	NO3	0.00	0.49	0.00	0.45	0.35	-0.15	0.40	-0.21	0.57	0.16
	NH4	0.03	0.41	0.02	0.29	0.25	0.05	0.43	0.04	0.31	0.19
Nationwide SF (satellite)	SO4	0.02	0.50	0.02	0.62	0.61	0.05	0.46	0.08	0.73	0.55
	NO3	0.26	0.59	0.24	0.55	0.35	0.07	0.44	0.10	0.63	0.16
	NH4	-0.17	0.40	-0.12	0.28	0.25	-0.15	0.41	-0.11	0.30	0.19
Scaled with state SF (AQS)	SO4	0.00	0.28	0.00	0.35	0.62	-0.04	0.24	-0.07	0.37	0.58
	NO3	0.00	0.38	0.00	0.35	0.60	-0.11	0.29	-0.15	0.41	0.53
	NH4	0.04	0.29	0.03	0.20	0.44	0.05	0.30	0.04	0.22	0.40
Scaled with state SF (satellite)	SO4	0.06	0.29	0.07	0.36	0.60	-0.01	0.22	-0.01	0.35	0.56
	NO3	0.14	0.44	0.13	0.41	0.60	0.03	0.31	0.04	0.44	0.60
	NH4	-0.03	0.27	-0.02	0.18	0.48	-0.02	0.30	-0.02	0.22	0.40
Closest 5 monitors (AQS)	SO4	-0.06	0.57	-0.08	-0.08	0.67	0.00	0.50	0.00	0.00	0.64
	NO3	0.41	0.71	0.37	0.37	0.58	0.35	0.61	0.49	0.49	0.41
	NH4	0.34	0.55	0.24	0.24	0.41	0.42	0.62	0.31	0.31	0.39
Grid cell (satellite)	SO4	0.02	0.11	0.03	0.13	0.93	0.04	0.09	0.07	0.15	0.88
	NO3	-0.02	0.18	-0.01	0.16	0.86	-0.01	0.14	-0.01	0.20	0.80
	NH4	-0.03	0.13	-0.02	0.09	0.82	-0.02	0.12	-0.02	0.09	0.84
Cities >350K pop boundary	SO4	0.02	0.15	0.03	0.03	0.71	0.01	0.14	0.02	0.02	0.70
	NO3	0.07	0.27	0.10	0.10	0.67	0.06	0.27	0.09	0.09	0.65
	NH4	0.03	0.21	0.02	0.02	0.67	0.04	0.20	0.03	0.03	0.68

Type of Analysis		Semi-urban					Rural				
Scaling type	Species	NMB	NME	MB	ME	R2	NMB	NME	MB	ME	R2
Not scaled	SO4	-0.43	0.45	-0.70	0.74	0.54	-0.53	0.63	-0.47	0.55	0.61
	NO3	-0.06	0.32	-0.07	0.35	0.52	0.61	0.91	0.27	0.40	0.46
	NH4	0.28	0.50	0.19	0.34	0.15	0.40	0.52	0.23	0.31	0.42
Nationwide SF (AQS)	SO4	0.11	0.37	0.18	0.60	0.53	-0.09	0.59	-0.08	0.52	0.61
	NO3	-0.13	0.32	-0.15	0.35	0.52	0.49	0.83	0.22	0.36	0.46
	NH4	-0.25	0.37	-0.17	0.25	0.15	-0.17	0.39	-0.10	0.23	0.42
Nationwide SF (satellite)	SO4	0.13	0.37	0.21	0.62	0.54	-0.08	0.59	-0.07	0.52	0.61
	NO3	0.09	0.35	0.10	0.38	0.52	0.87	1.12	0.38	0.49	0.46
	NH4	-0.39	0.43	-0.27	0.30	0.15	-0.34	0.44	-0.20	0.26	0.42
Scaled with state SF (AQS)	SO4	-0.08	0.24	-0.13	0.39	0.56	0.10	0.38	0.09	0.33	0.63
	NO3	-0.20	0.39	-0.22	0.43	0.45	0.36	0.66	0.16	0.29	0.60
	NH4	-0.18	0.24	-0.12	0.17	0.57	-0.05	0.25	-0.03	0.14	0.73
Scaled with state SF (satellite)	SO4	-0.02	0.25	-0.04	0.41	0.47	0.18	0.42	0.16	0.37	0.58
	NO3	-0.09	0.43	-0.10	0.47	0.38	0.57	0.84	0.25	0.37	0.46
	NH4	-0.21	0.27	-0.15	0.19	0.52	-0.12	0.25	-0.07	0.15	0.71
Scaled with state SF (satellite)	SO4	0.11	0.43	0.17	0.17	0.72	-0.22	0.72	-0.19	-0.19	0.64
	NO3	0.34	0.73	0.38	0.38	0.51	0.60	1.02	0.27	0.27	0.77
	NH4	-0.01	0.30	-0.01	-0.01	0.47	0.09	0.24	0.05	0.05	0.83
Grid cell (satellite)	SO4	-0.01	0.17	-0.01	0.28	0.72	-0.01	0.11	-0.01	0.10	0.95
	NO3	-0.16	0.25	-0.17	0.28	0.54	0.01	0.26	0.00	0.11	0.82
	NH4	-0.07	0.22	-0.05	0.15	0.61	-0.02	0.14	-0.01	0.08	0.87
Cities >350K pop boundary	SO4	0.02	0.15	0.03	0.03	0.76	-	-	-	-	-
	NO3	0.24	0.33	0.20	0.20	0.58	-	-	-	-	-
	NH4	-0.22	0.34	-0.14	-0.14	0.36	-	-	-	-	-

Table S3. Model performance statistics: Normalized Mean Bias (NMB, %), Normalized Mean Error (NME, %), Mean Bias (MB, μg/m^3), Mean Error (ME, μg/m^3), and coefficient of determination (R^2) for grid-scaling methodology evaluated against ground-level monitors. We present each statistic for the InMAP domain as a whole (overall) as well as aggregated into urban, semi-urban, and rural place designations.

Type of Analysis		Overall					Urban				
Scaling type	Species	NMB	NME	MB	ME	R2	NMB	NME	MB	ME	R2
Unscaled	SO4	-0.49	0.51	-0.71	0.74	0.59	-0.50	0.51	-0.72	0.75	0.60
	NO3	-0.14	0.50	-0.17	0.60	0.11	-0.21	0.48	-0.28	0.63	0.10
	NH4	1.08	1.13	0.65	0.69	0.33	1.16	1.21	0.73	0.76	0.33
Nationwide SF (monitor)	SO4	-0.01	0.50	-0.02	0.72	0.59	-0.02	0.52	-0.04	0.76	0.60
	NO3	-0.20	0.50	-0.24	0.60	0.11	-0.27	0.48	-0.36	0.63	0.10
	NH4	0.23	0.50	0.14	0.31	0.33	0.28	0.53	0.18	0.33	0.33
Nationwide SF (satellite)	SO4	0.00	0.50	0.01	0.73	0.59	-0.01	0.53	-0.01	0.77	0.60
	NO3	0.00	0.54	0.00	0.64	0.11	-0.09	0.50	-0.11	0.65	0.10
	NH4	-0.01	0.43	-0.01	0.26	0.33	0.03	0.44	0.02	0.28	0.33
Scaled with state SF (monitor)	SO4	-0.05	0.25	-0.08	0.36	0.55	-0.07	0.25	-0.10	0.37	0.56
	NO3	-0.19	0.34	-0.22	0.41	0.46	-0.23	0.34	-0.30	0.45	0.45
	NH4	0.06	0.29	0.03	0.18	0.47	0.06	0.29	0.04	0.18	0.48
Scaled with state SF (satellite)	SO4	0.00	0.23	0.00	0.34	0.54	-0.01	0.23	-0.01	0.34	0.54
	NO3	0.00	0.31	0.00	0.37	0.57	-0.03	0.30	-0.03	0.39	0.56
	NH4	0.00	0.26	0.00	0.16	0.55	0.01	0.26	0.01	0.16	0.54
Closest 5 monitors (monitor)	SO4	-0.12	0.54	-0.16	-0.16	0.63	-0.11	0.55	-0.16	-0.16	0.64
	NO3	0.17	0.58	0.19	0.19	0.44	0.13	0.54	0.16	0.16	0.37
	NH4	0.55	0.75	0.32	0.32	0.49	0.72	0.86	0.45	0.45	0.47
Grid cell (satellite)	SO4	0.00	0.00	0.00	0.00	1.00	0.00	0.00	0.00	0.00	1.00
	NO3	0.00	0.00	0.00	0.00	1.00	0.00	0.00	0.00	0.00	1.00
	NH4	0.00	0.00	0.00	0.00	1.00	0.00	0.00	0.00	0.00	1.00
Cities >350K pop boundary	SO4	0.00	0.15	0.00	0.00	0.73	0.00	0.15	0.00	0.00	0.72
	NO3	0.00	0.27	0.00	0.00	0.64	0.00	0.27	0.00	0.00	0.64
	NH4	0.00	0.19	0.00	0.00	0.74	0.00	0.19	0.00	0.00	0.74

Type of Analysis		Semi-urban					Rural				
Scaling type	Species	NMB	NME	MB	ME	R2	NMB	NME	MB	ME	R2
Unscaled	SO4	-0.44	0.47	-0.66	0.71	0.56	-0.48	0.51	-0.66	0.70	0.60
	NO3	0.20	0.53	0.17	0.46	0.27	0.47	0.71	0.33	0.51	0.33
	NH4	0.63	0.74	0.36	0.42	0.22	0.73	0.83	0.36	0.41	0.30
Nationwide SF (AQS)	SO4	0.09	0.42	0.13	0.64	0.56	0.00	0.40	0.00	0.54	0.60
	NO3	0.11	0.50	0.09	0.43	0.27	0.35	0.64	0.25	0.46	0.33
	NH4	-0.03	0.40	-0.02	0.22	0.22	0.02	0.40	0.01	0.20	0.30
Nationwide SF (satellite)	SO4	0.11	0.43	0.16	0.65	0.56	0.02	0.40	0.02	0.55	0.60
	NO3	0.39	0.64	0.34	0.56	0.27	0.70	0.88	0.50	0.63	0.33
	NH4	-0.22	0.41	-0.13	0.23	0.22	-0.18	0.39	-0.09	0.19	0.30
Scaled with state SF (AQS)	SO4	0.00	0.23	0.00	0.35	0.48	0.00	0.25	-0.01	0.34	0.54
	NO3	0.00	0.30	0.00	0.26	0.57	0.14	0.36	0.10	0.26	0.62
	NH4	-0.02	0.28	-0.01	0.16	0.42	0.10	0.32	0.05	0.16	0.41
Scaled with state SF (satellite)	SO4	0.03	0.22	0.04	0.33	0.51	0.03	0.24	0.03	0.33	0.56
	NO3	0.08	0.28	0.07	0.25	0.64	0.24	0.38	0.17	0.27	0.65
	NH4	-0.09	0.24	-0.05	0.13	0.55	-0.01	0.25	-0.01	0.12	0.56
Closest 5 monitors (AQS)	SO4	-0.01	0.49	-0.01	-0.01	0.63	-0.20	0.54	-0.25	-0.25	0.62
	NO3	0.44	0.72	0.35	0.35	0.62	0.33	0.76	0.21	0.21	0.67
	NH4	0.18	0.45	0.10	0.10	0.46	0.02	0.43	0.01	0.01	0.58
Grid cell (satellite)	SO4	0.00	0.00	0.00	0.00	1.00	0.00	0.00	0.00	0.00	1.00
	NO3	0.00	0.00	0.00	0.00	1.00	0.00	0.00	0.00	0.00	1.00
	NH4	0.00	0.00	0.00	0.00	1.00	0.00	0.00	0.00	0.00	1.00
Cities >350K pop boundary	SO4	0.04	0.14	0.06	0.06	0.77	-	-	-	-	-
	NO3	0.06	0.23	0.05	0.05	0.73	-	-	-	-	-
	NH4	-0.09	0.19	-0.05	-0.05	0.74	-	-	-	-	-

Table S4. Model performance statistics: Normalized Mean Bias (NMB, %), Normalized Mean Error (NME, %), Mean Bias (MB, μg/m^3), Mean Error (ME, μg/m^3), and coefficient of determination (R^2) for each scaling methodology and $PM_{2.5}$ species evaluated against satellite-derived data. We present each statistic for the InMAP domain as a whole (overall) as well as aggregated into urban, semi-urban, and rural place designations.

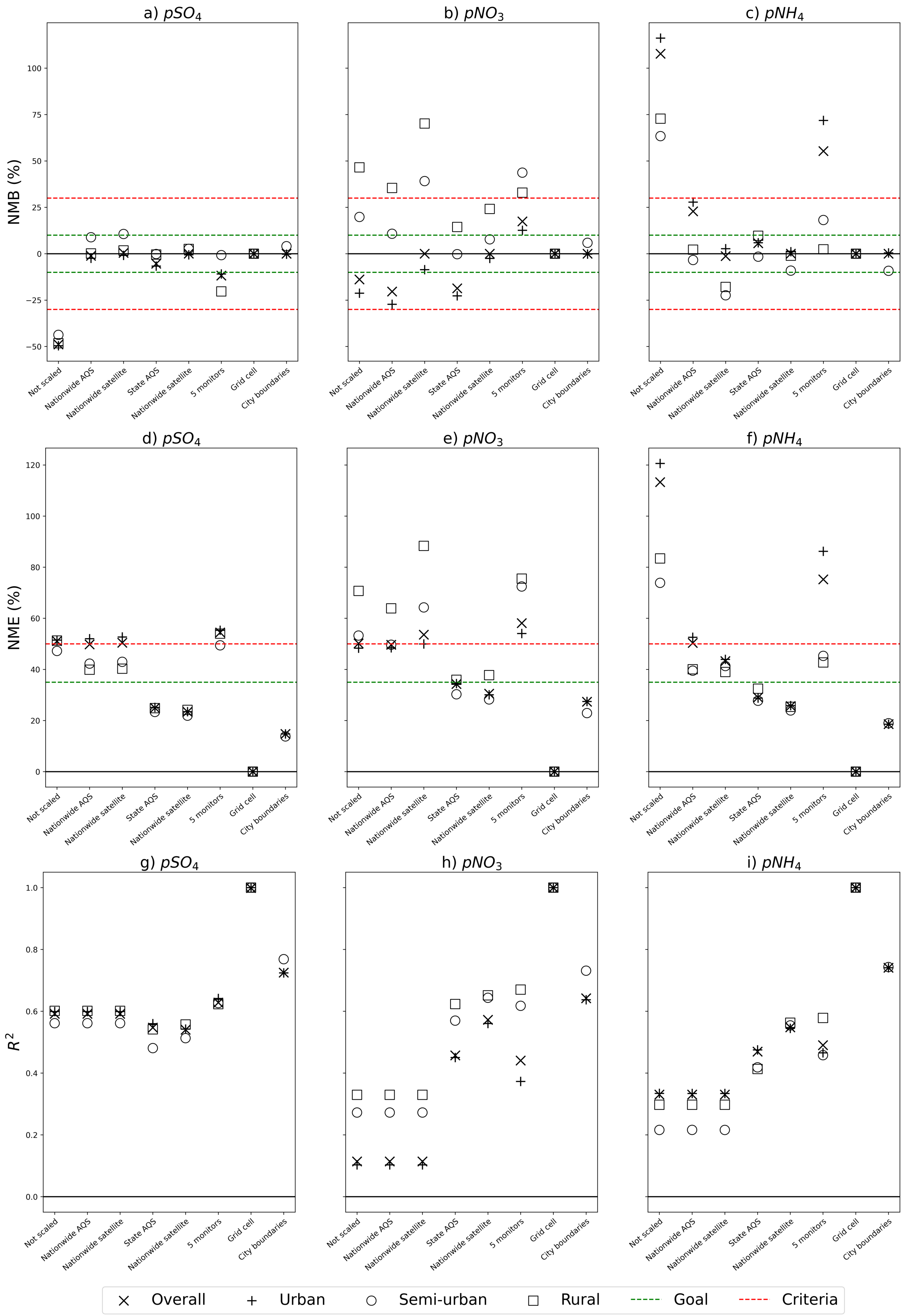

Figure S4. Model performance statistics Normalized Mean Bias (NMB, a-c), Normalized Mean Error (NME, d-f), and coefficient of determination (R^2, g-i) for overall model domain, urban, semi-urban, and rural grid cells for each scaling methodology, calculated with satellite-derived data. From left to right the x-axis, we plot unscaled InMAP predictions and the seven scaling approaches that apply scaling factors determined by nationwide AQS, nationwide satellite, state AQS, state satellite, 5 monitors, grid cell, and city boundaries. NMB and NME graphs also have goal and criteria benchmarks mapped, as recommended by Emery et al. (2017).

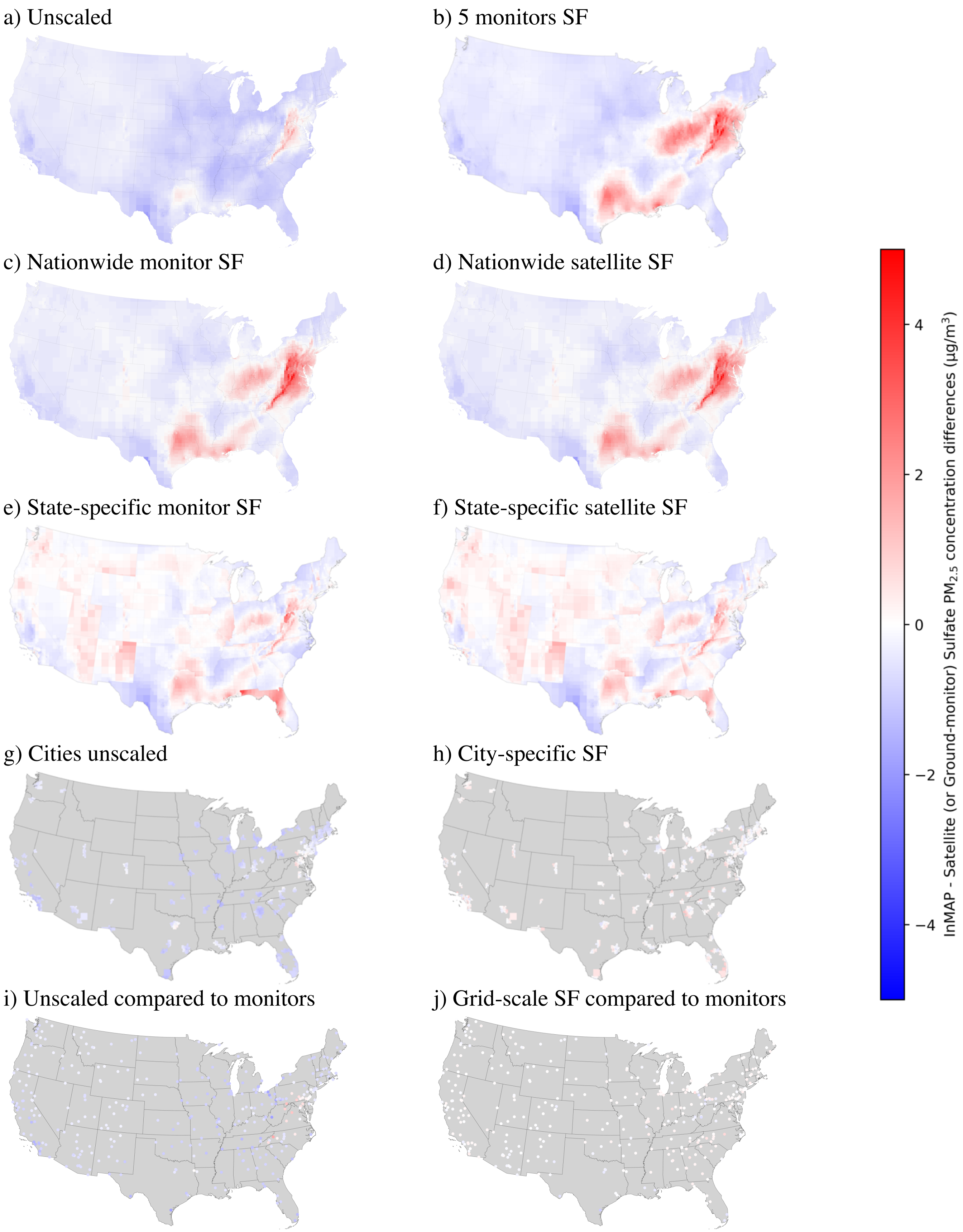

Figure S5. The difference between InMAP predictions and satellite observations for particulate sulfate for a) unscaled, b) 5 monitors SF, c) nationwide monitor SF, d) nationwide satellite SF, e) state-specific monitor SF, f) state-specific satellite SF, g) unscaled for large U.S. cities, h) city-specific SF for large U.S. cities, i) unscaled compared to ground-level monitors, j) grid-scaled compared to ground-level monitors.

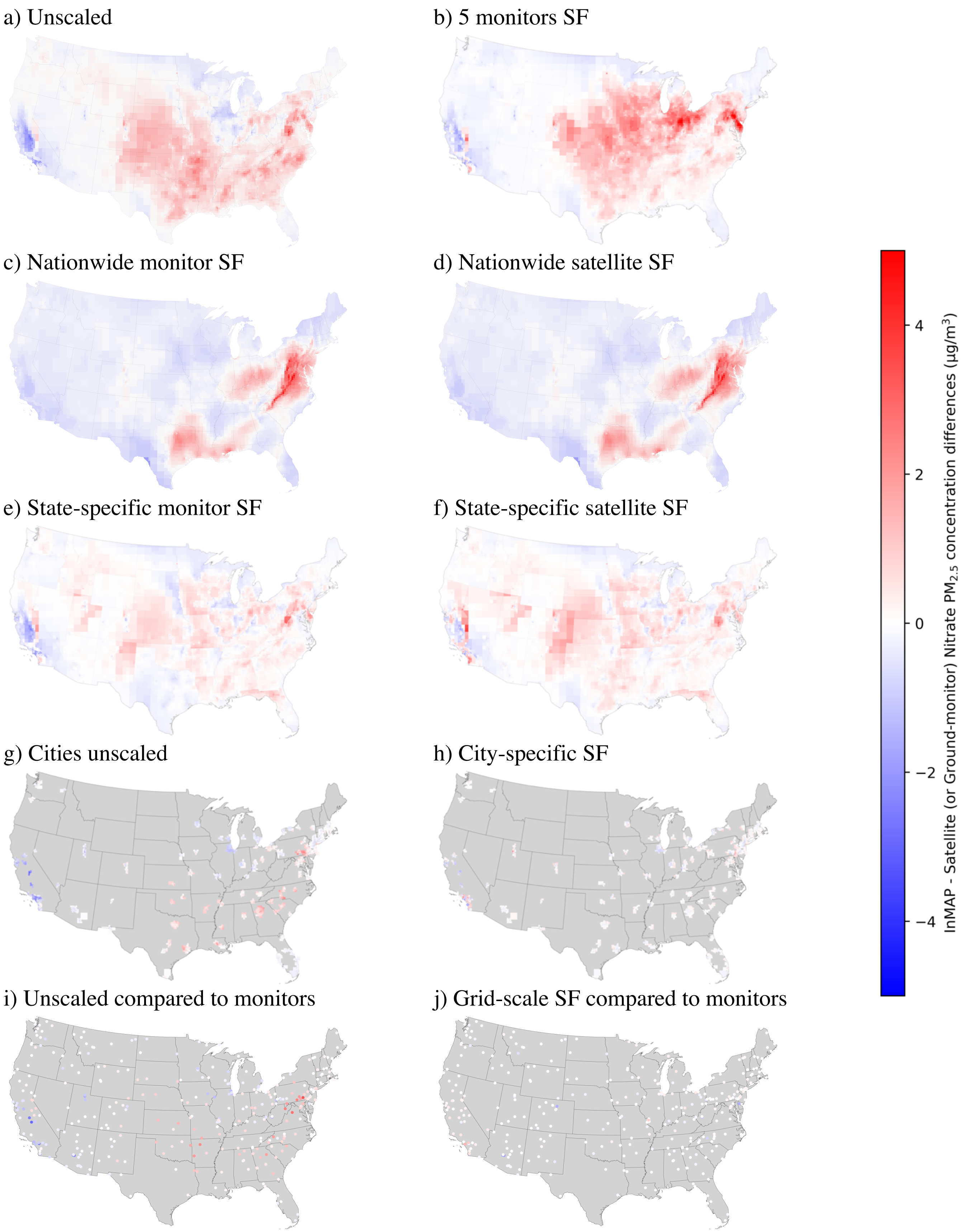

Figure S6. The difference between InMAP predictions and satellite observations for particulate nitrate for a) unscaled, b) 5 monitors SF, c) nationwide monitor SF, d) nationwide satellite SF, e) state-specific monitor SF, f) state-specific satellite SF, g) unscaled for large U.S. cities, h) city-specific SF for large U.S. cities, i) unscaled compared to ground-level monitors, j) grid-scaled compared to ground-level monitors.

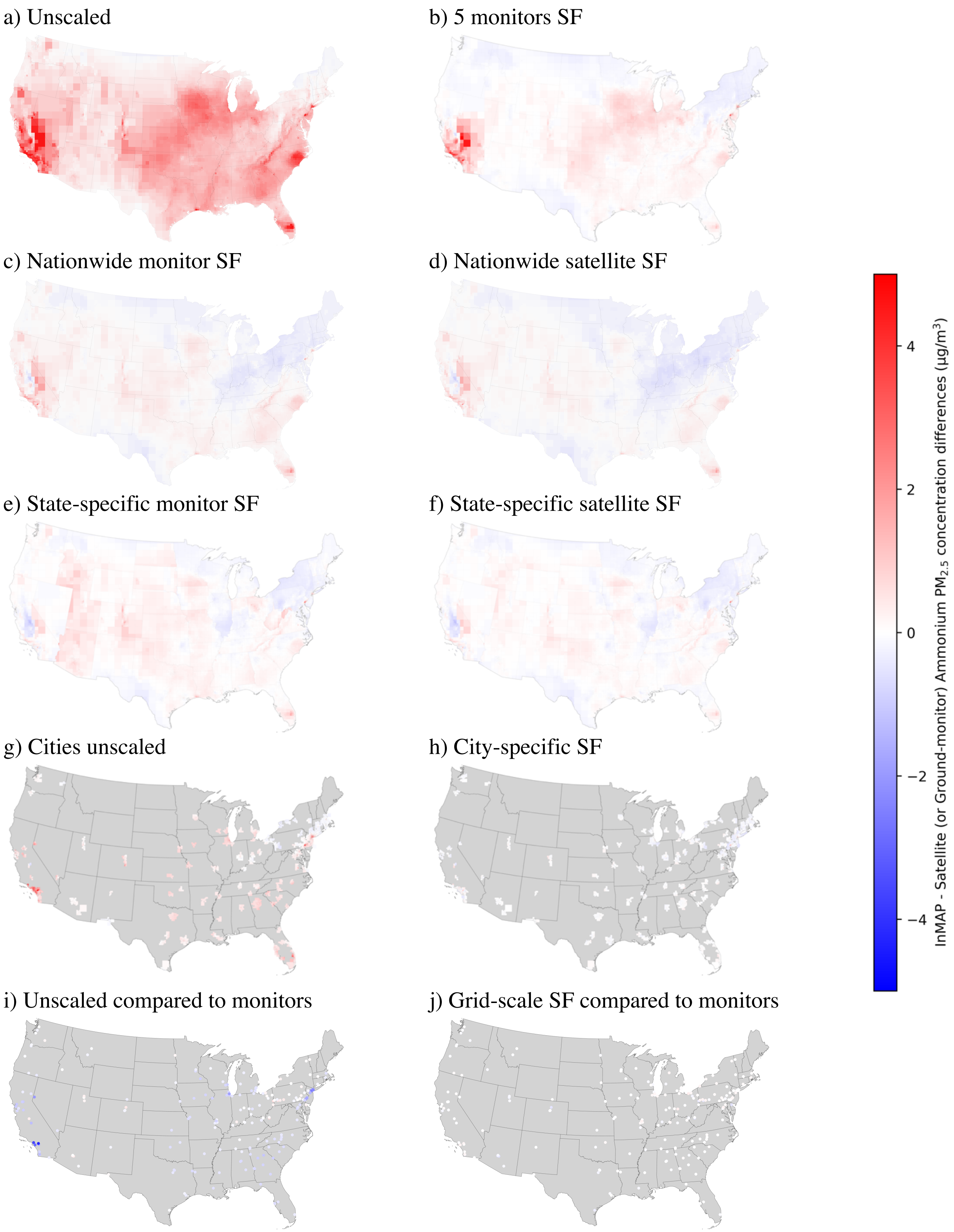

Figure S7. The difference between InMAP predictions and satellite observations for particulate ammonium for a) unscaled, b) 5 monitors SF, c) nationwide monitor SF, d) nationwide satellite SF, e) state-specific monitor SF, f) state-specific satellite SF, g) unscaled for large U.S. cities, h) city-specific SF for large U.S. cities, i) unscaled compared to ground-level monitors, j) grid-scaled compared to ground-level monitors.

City or group of cities	Percent (%) grid cells improved pSO4 predictions	Percent (%) grid cells improved pNO3 predictions	Percent (%) grid cells improved pNH4 predictions	Comparison data
Chicago, IL	91	86	97	Satellite data
Detroit, MI	100	83	97	
El Paso, TX	99	79	97	
Kansas City, MO	100	69	100	
Las Vegas, NV	76	67	100	
Los Angeles--Long Beach--Anaheim, CA	97	83	97	
Louisville, KY	98	80	40	
New Orleans, LA	100	87	96	
New York--Newark, NY--NJ	85	42	84	
Phoenix--Mesa, AZ	99	87	88	
Pittsburgh, PA	100	80	60	
St. Louis, MO	100	74	95	
All EJ cities of concern	93	71	91	
All city-scaled cities	90	72	88	
All city-scaled cities	58	63	42	Ground-monitor data

Table S5. For each city and collection of cities the percentage of grid cells for which scaling improves alignment with observational values (satellite-derived or ground-monitor data).

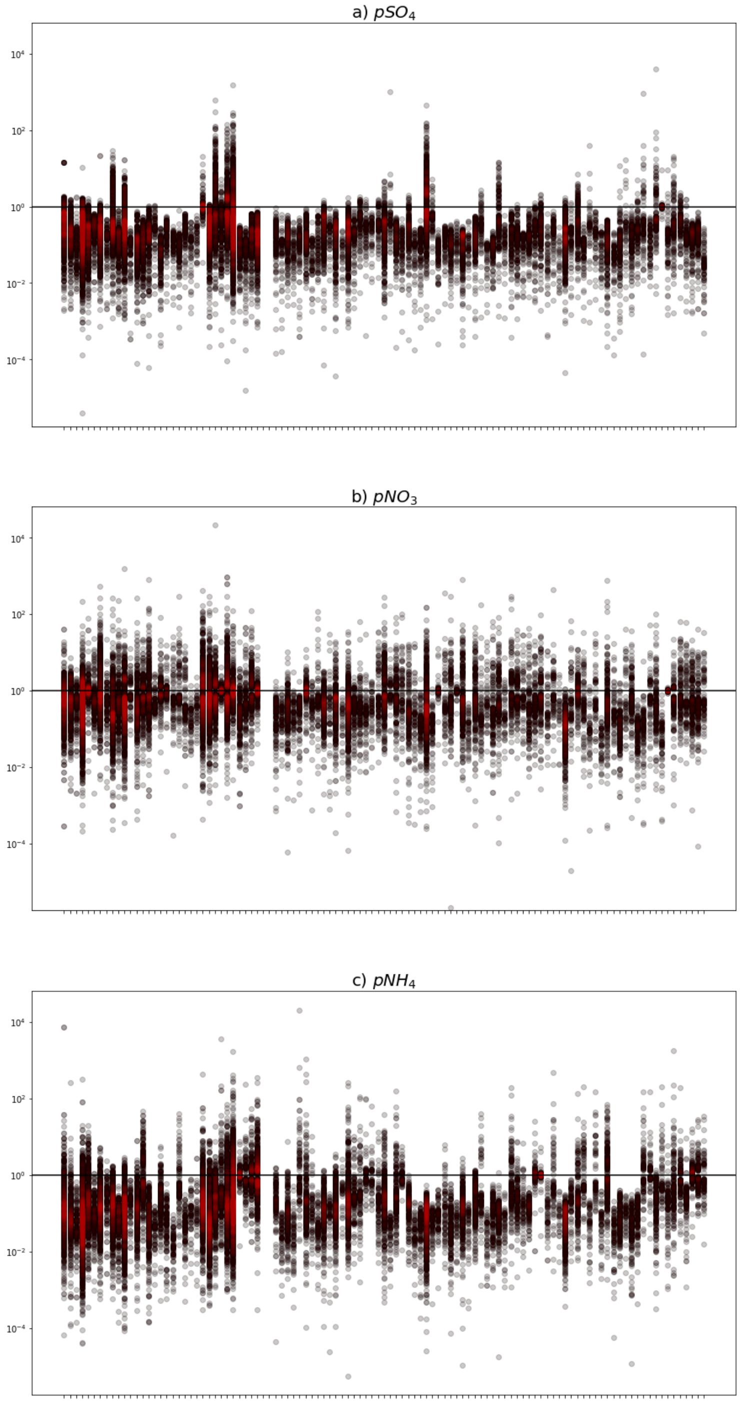

Figure S8. Improvement ratio for all grid-cells in city-scaled cities as calculated with satellite-derived data for a) pSO_4, b) pNO_3, and c) pNH_4. Red points indicate the highest density of data points. Each discrete column on the x-axis represents one city.

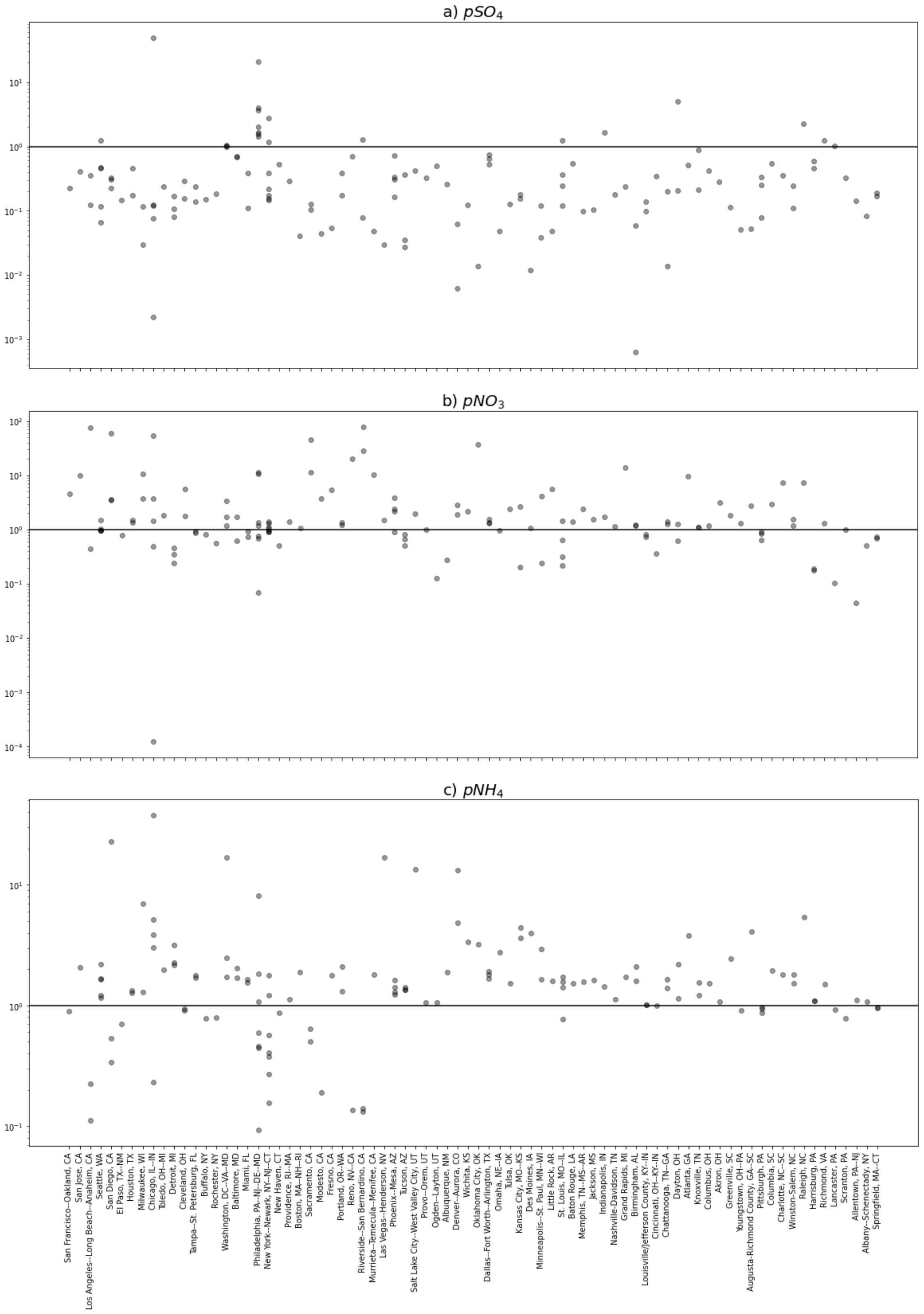

Figure S9. Improvement ratio for all grid-cells in city-scaled cities as calculated with ground-monitor data for a) pSO_4, b) pNO_3, and c) pNH_4.

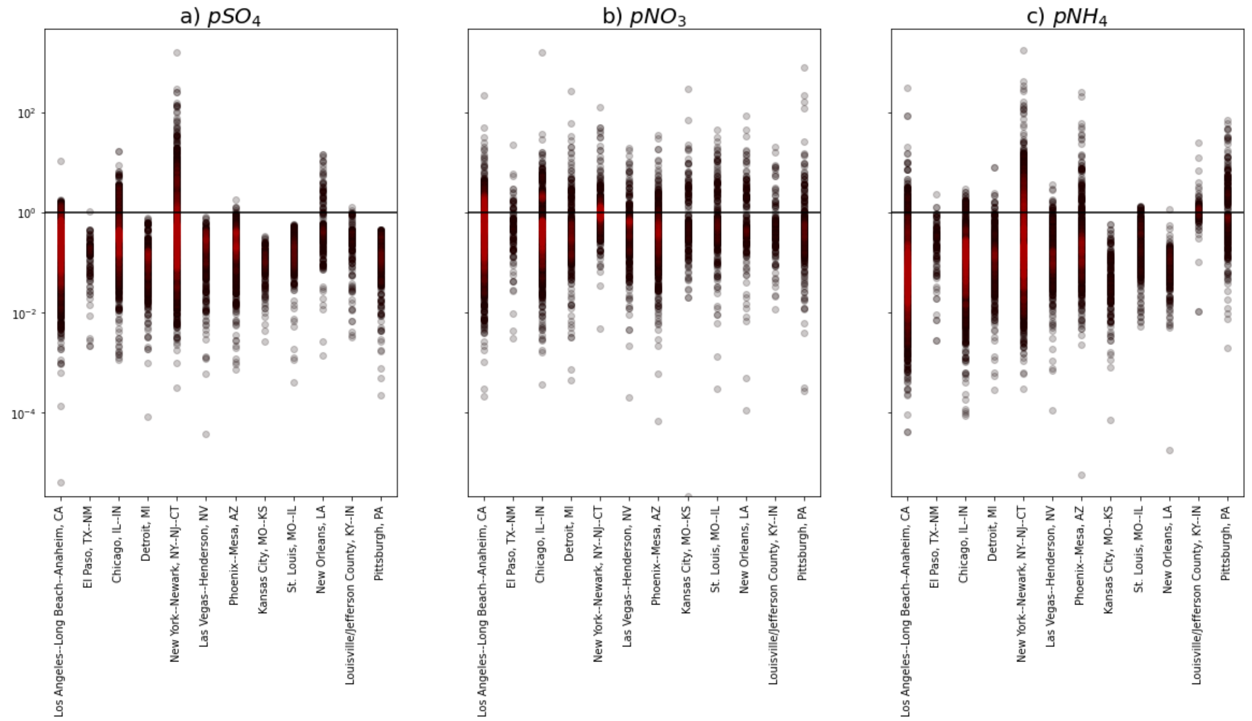

Figure S10. Improvement ratio for twelve illustrative cities as calculated with satellite-derived data for a) pSO_4, b) pNO_3, and c) pNH_4. Red points indicate the highest density of data points.

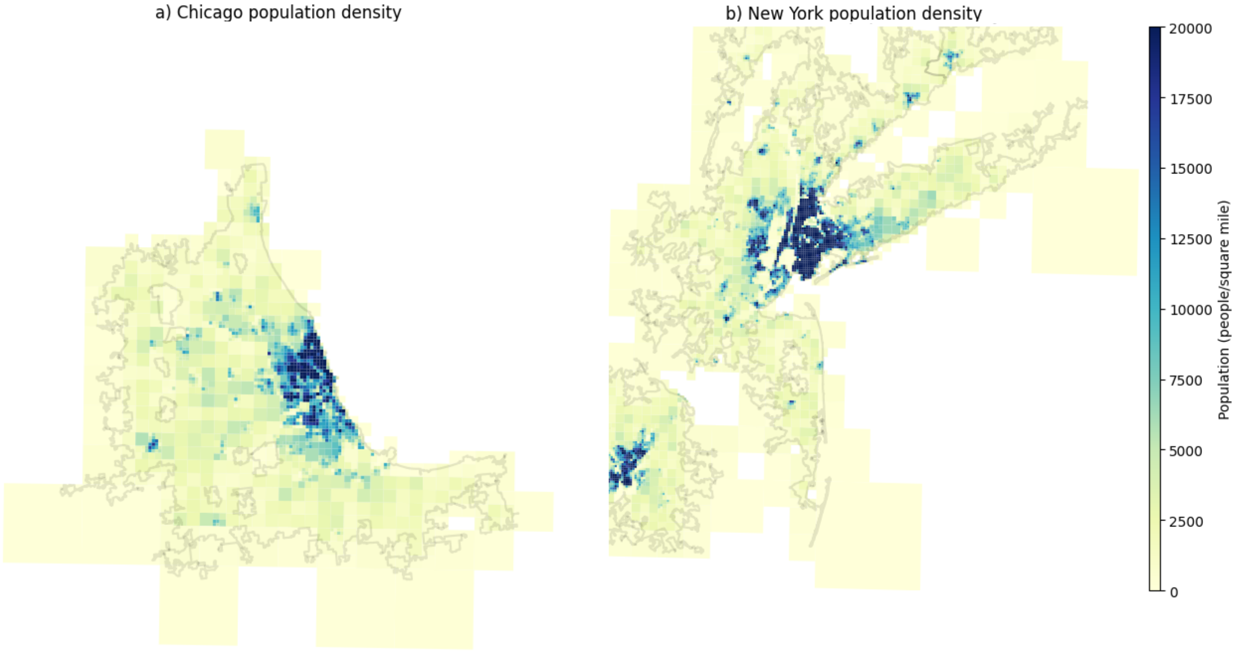

Figure S11. Population density (people/mi^2) for a) Chicago, IL and b) Newark-New York, NJ-NY.

	Agriculture	Area Fugitive Dust	Non-EGU point	EGU point	Non-road transportation	On-road transportation	Fires	Oil and Gas	Rail	Residential Wood Combustion	Non-point
Nationwide	-4.2	-0.3	2.7	3.9	0.3	0.2	0.0	0.0	0.1	-0.2	-2.5
Chicago, IL	-6.3	-0.7	1.9	5.6	0.3	0.7	-0.2	0.1	0.3	-0.2	-1.6
Detroit, MI	-5.2	-0.9	2.2	8.1	-0.3	-0.8	-0.2	0.1	0.1	-0.8	-2.4
El Paso, TX	0.8	-7.4	3.3	11.5	-1.6	-3.0	0.1	4.7	-0.1	-0.7	-7.7
Kansas City, MO	-6.5	-0.2	1.8	8.4	-0.4	-1.9	-0.2	-0.1	-0.4	-0.1	-0.3
Las Vegas, NV	-5.8	-1.1	2.3	1.7	0.3	2.4	0.8	0.2	0.1	-0.3	-0.7
Los Angeles--Long Beach--Anaheim, CA	-3.5	-0.1	8.2	0.0	2.2	5.6	0.2	0.1	0.8	0.0	-13.5
Louisville, KY	-0.8	-0.5	-0.3	3.4	-0.2	0.1	-0.2	0.0	0.0	-0.3	-1.3
New Orleans, LA	-2.4	0.1	0.6	3.1	0.0	-1.4	-0.3	0.0	-0.1	0.0	0.4
New York--Newark, NY--NJ	-1.9	0.4	0.6	3.5	1.0	-0.5	0.0	0.1	0.0	0.4	-3.6
Phoenix--Mesa, AZ	-1.3	-2.7	4.4	2.4	-0.6	0.9	0.2	0.2	0.1	-0.6	-3.0
Pittsburgh, PA	-0.3	-0.6	0.5	5.7	-0.5	-0.6	-0.1	-0.1	-0.1	-0.9	-2.9
St. Louis, MO	-3.2	-1.5	0.4	7.7	-0.3	-0.6	-0.6	0.1	0.0	-0.5	-1.5

Table S6. Percent change (%) in pollution contribution from all InMAP emissions sources between unscaled and scaled (grid cell and city boundaries) InMAP predictions for the contigous U.S. and the twelve illustrative cities.

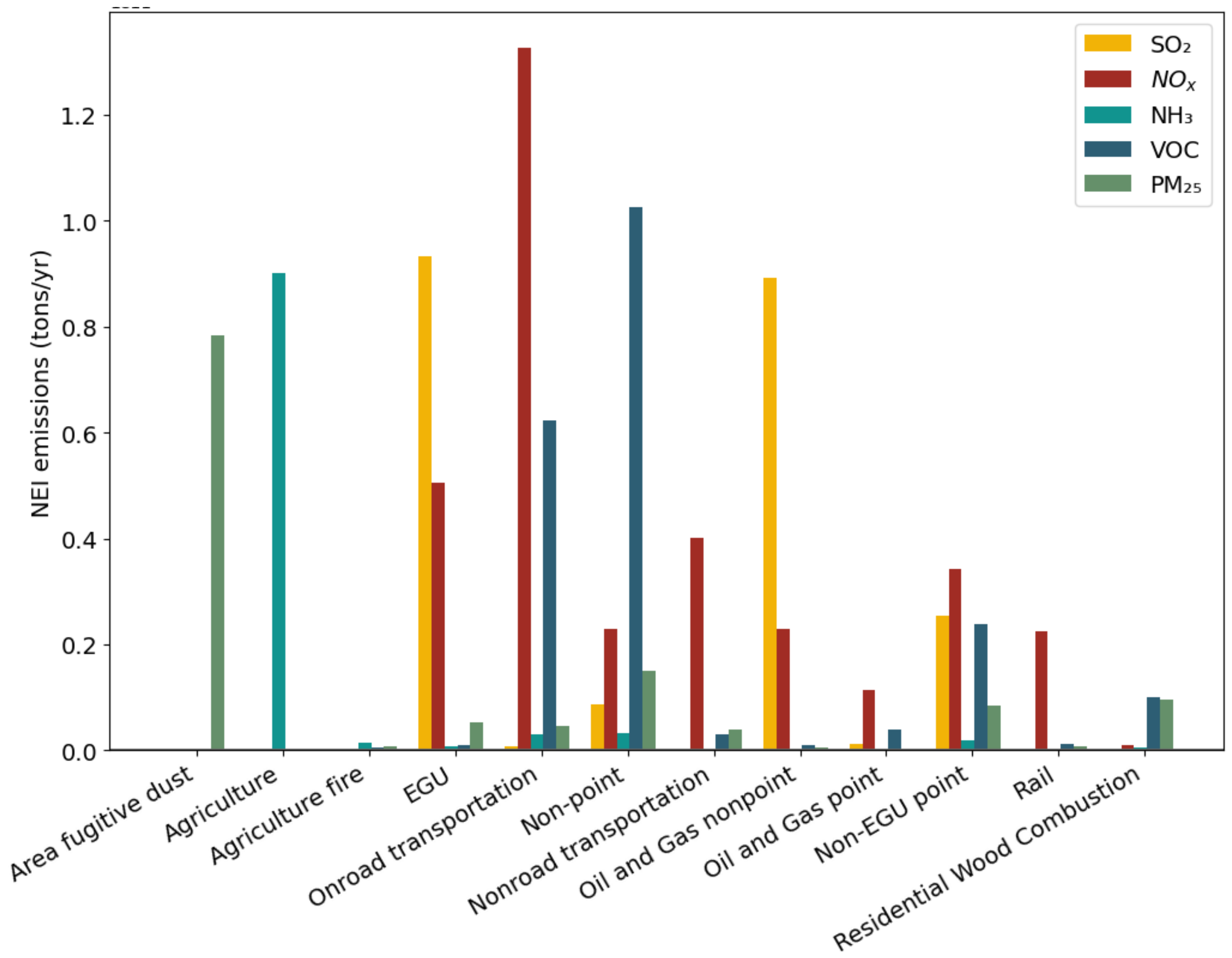

Figure S12. Pollutant emissions for InMAP source input files.
